# Dual Effect of Combined Metformin and 2-Deoxy-D-Glucose Treatment on Mitochondrial Biogenesis and PD-L1 Expression in Triple-Negative Breast Cancer Cells

**DOI:** 10.3390/cancers14051343

**Published:** 2022-03-05

**Authors:** Jernej Repas, Mateja Zupin, Maja Vodlan, Peter Veranič, Boris Gole, Uroš Potočnik, Mojca Pavlin

**Affiliations:** 1 Institute of Biophysics, Faculty of Medicine, University of Ljubljana, SI-1000 Ljubljana, Slovenia; jernej.repas@mf.uni-lj.si (J.R.); vodlan.maja@gmail.com (M.V.); 2 Center for Human Molecular Genetics and Pharmacogenomics, Faculty of Medicine, University of Maribor, SI-2000 Maribor, Slovenia; mateja.zupin@um.si (M.Z.); boris.gole@um.si (B.G.); uros.potocnik@um.si (U.P.); 3 Institute of Cell Biology, Faculty of Medicine, University of Ljubljana, SI-1000 Ljubljana, Slovenia; peter.veranic@mf.uni-lj.si; 4 Laboratory for Biochemistry, Molecular Biology and Genomics, University of Maribor, SI-2000 Maribor, Slovenia; 5 Department for Science and Research, University Medical Centre Maribor, SI-2000 Maribor, Slovenia; 6 Group for Nano- and Biotechnological Applications, Faculty of Electrical Engineering, University of Ljubljana, SI-1000 Ljubljana, Slovenia

**Keywords:** metformin, 2-deoxy-D-glucose, triple-negative breast cancer, T cells, mitochondrial biogenesis, protein N-glycosylation, ER stress, AMPK, PD-1/PD-L1 axis, anchorage-independence

## Abstract

**Simple Summary:**

Metformin and 2-deoxy-D-glucose are metabolic drugs with multiple and incompletely understood anti-cancer effects. Their combination can cause breast cancer cell detachment from the growth surface. Mitochondria are important for detached cell survival and metastasis, but how metformin and 2DG affect cancer mitochondria is largely unknown. We found that metformin and 2-deoxy-D-glucose together increased mitochondrial mass in triple-negative breast cancer cells due to the enlargement of mitochondria, and did not decrease their degradation. Both the reduction in protein-attached sugars and reduced ATP production seemed to be involved in triggering the process. Metformin and 2-deoxy-D-glucose can reduce immune checkpoint PD-L1 levels, responsible for immune escape. We found that the reduction in protein-attached sugars caused by metformin and 2DG also reduced PD-L1 levels on breast cancer cells and its partner receptor PD-1 on activated T cells. While the activation of T cells was reduced, they mostly maintained their effector functions. Metformin and 2-deoxy-D-glucose could therefore potentially improve anti-cancer immunity.

**Abstract:**

Metformin and 2-deoxy-D-glucose (2DG) exhibit multiple metabolic and immunomodulatory anti-cancer effects, such as suppressed proliferation or PD-L1 expression. Their combination or 2DG alone induce triple-negative breast cancer (TNBC) cell detachment, but their effects on mitochondria, crucial for anchorage-independent growth and metastasis formation, have not yet been evaluated. In the present study, we explored the effects of metformin, 2DG and their combination (metformin + 2DG) on TNBC cell mitochondria in vitro. Metformin + 2DG increased mitochondrial mass in TNBC cells. This was associated with an increased size but not number of morphologically normal mitochondria and driven by the induction of mitochondrial biogenesis rather than suppressed mitophagy. 2DG and metformin + 2DG strongly induced the unfolded protein response by inhibiting protein N-glycosylation. Together with adequate energy stress, this was one of the possible triggers of mitochondrial enlargement. Suppressed N-glycosylation by 2DG or metformin + 2DG also caused PD-L1 deglycosylation and reduced surface expression in MDA-MB-231 cells. PD-L1 was increased in low glucose and normalized by both drugs. 2DG and metformin + 2DG reduced PD-1 expression in Jurkat cells beyond the effects on activation, while cytokine secretion was mostly preserved. Despite increasing mitochondrial mass in TNBC cells, metformin and 2DG could therefore potentially be used as an adjunct therapy to improve anti-tumor immunity in TNBC.

## 1. Introduction

The emerging importance of energy metabolism in cancer has led to the investigation of metabolic drugs as potential anti-cancer therapies [1,2]. One of the most promising of these is the antidiabetic drug metformin, which has been associated with decreased incidence of several cancer types, including breast cancer [3]. While the use of metformin as an anti-cancer agent is now being evaluated in numerous clinical trials [4,5], there is still ongoing debate on its mechanisms of action, which have not been fully explained. Metformin acts systemically by inhibiting gluconeogenesis in the liver and improving glycemic control, but can also act directly on cancer cells by inhibiting complex I of the respiratory electron transport chain (ETC) [6], which leads to AMPK activation [7]. However, metformin can act also independently of AMPK activation, for example, by inhibiting mTOR signaling and inducing cell cycle arrest [8,9], and by its direct effects on cancer cells metabolism, suppressing biosynthetic reactions that rely on reduced cofactors and reduced nucleotide levels [9,10,11].

Despite the well-characterized effects of metformin on the ETC, its effect on mitochondrial dynamics and its anticancer effects remain poorly understood. Energy stress and AMPK activation are known to induce mitochondrial biogenesis via peroxisome proliferator-activated receptor gamma coactivator 1-alpha (PGC-1α) in skeletal muscle cells and hepatocytes [12,13,14]. The induction of mitochondrial biogenesis could importantly influence the response of cancer cells to metabolic drugs such as metformin, as additional respiratory capacity could render cells more resilient to energy stress, as has been demonstrated for breast cancer’s resistance to metformin in vivo [15]. In addition, mitochondrial function was shown to be important in the process of cell detachment, anchorage-independent growth [16,17,18] and metastasis formation [19]. However, few studies to date have evaluated the effect of metformin on mitochondrial biogenesis in cancer cells.

The effect of metformin on proliferation is strongly dependent on the intrinsic characteristics of cancer cells which are often capable of compensating inhibited oxidative phosphorylation with increased glycolysis. To overcome this adaptation, synergistic action with glycolysis inhibitors, such as 2-deoxy-D-glucose (2DG), was explored [2,20,21,22,23]. 2DG is a competitive inhibitor of hexokinase and phosphoglucose isomerase [24]. In addition, 2DG was shown to selectively inhibit protein N-glycosylation, leading to ER stress and unfolded protein response (UPR), which can also contribute to cancer cell apoptosis [25,26,27,28,29]. The combination of metformin and 2DG has been shown to synergistically suppress cancer cell proliferation [20,22] and decrease angiogenesis [23]. Previously, we have shown that the combination of metformin and in vivo achievable concentration of 2DG induces detachment of MDA-MB-231 triple-negative breast cancer (TNBC) cells and anchorage-independent growth [30]. As anchorage-independent growth is closely linked to mitochondrial metabolism and biogenesis [17,31,32], treatment with metformin and 2DG could induce mitochondrial biogenesis; however, this question has not yet been investigated.

In addition to mitochondrial biogenesis, the homeostasis of mitochondria is also dependent on mitophagy [33]. The induction of autophagy is generally detrimental to fast proliferation; however, it can help cancer cells to survive in environments where nutrients are scarce [34]. 2DG has been shown to induce autophagy through AMPK activation and ER stress [35,36]. While the combination of metformin and 2DG can block autophagy [20], their effect on mitophagy is yet to be thoroughly investigated.

Emerging evidence has also suggested that metabolic drugs including metformin can improve the anti-tumor immune response [37,38,39,40]. Metformin has been shown to ameliorate hypoxia in the tumor microenvironment, improving cytotoxic T cell function in the context of anti-PD-1 therapy [41]. Additionally, both metformin and 2DG have been shown to impact programmed death ligand 1 (PD-L1) glycosylation and degradation in breast cancer cells [42,43,44]; however, the effect of their combination on PD-L1 expression has not yet been studied. The effect of metformin and 2DG are also importantly impacted by nutrient availability [41,45,46]. Nevertheless, the effect of metformin and 2DG on mitochondrial biogenesis and PD-L1 expression as a function of glucose availability has not yet been explored. When used in vivo, metabolic drugs also come into contact with tumor-infiltrating T cells, and so it is important to explore their effects on T cells as well [47]. Mitochondrial mass is one of the key indicators of T-cell metabolic fitness required for successful anti-tumor response [48]; however, the effect of metformin and 2DG on T-cell mitochondria has not yet been studied. Likewise, the effect of metformin and 2DG on PD-1 expression has not yet been thoroughly explored.

In the present study, we explored the effect of metformin, 2DG and their combination on mitochondrial mass in MDA-MB-231 and BT-549 TNBC cells. We show that the combined treatment with metformin and 2DG increased mitochondrial mass by increasing mitochondria size. The induction of unfolded protein response (UPR) was observed via inhibited protein N-glycosylation for high dose 2DG or combined metformin + 2DG treatment, indicating a potential role in increasing mitochondrial size. We demonstrated that suppressed protein N-glycosylation by metformin and low 2DG also reduced PD-L1 expression in MDA-MB-231 cells and PD-1 expression on Jurkat cells used as a model T cells, while partially maintaining their effector function, suggesting metformin and 2DG treatment as a potential adjunct therapy in the context of cancer immunotherapy.

## 2. Results

### 2.1. The Combined Treatment with Metformin and 2DG Increases Mitochondrial Mass in TNBC Cells

We have previously shown that combined treatment with metformin and 0.6 mM 2DG induces the detachment of MDA-MB-231 breast cancer cells [30]. However, the metabolic adaptations and processes that lead to detachment and survival in anchorage-independent growth remain poorly understood. Among other alterations, mitochondrial metabolism and biogenesis was shown to help cells to adapt to the energetic stress and avoid anoikis [16,17,32].

**Figure 1:**
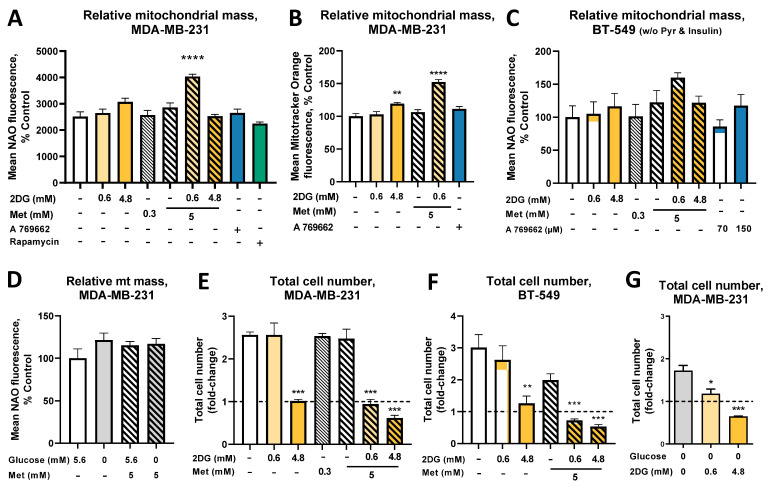
The effect of metformin and 2DG on mitochondrial mass. The cells were treated with 5 mM metformin and/or 0.6 mM 2DG or 4.8 mM 2DG for 72 h with daily medium change in medium supplemented with (**A**–**C**,**E**,**F**) 5.6 mM or (**D**,**G**) 0 mM glucose. The mitochondrial content was determined by NAO (**A**,**C**,**D**) or Mitotracker Orange (**B**) staining and flow cytometry. Total cell number (expressed as fold change over starting cell number, including detached cells) was determined with Trypan blue staining and direct cell counting (**E**–**G**). Mean ± SEM is shown for three (**B**–**G**) or four (**A**) independent experiments. Data is color-coded according to treatment (orange for 2DG, blue for A 769662, green for rapamycin and hatching for metformin). * *p* < 0.05, ** *p* < 0.01, *** *p* < 0.001, **** *p* < 0.0001 vs. control as determined by ANOVA with Dunnett’s post hoc test.

To quantify the effect of metformin and 2DG on mitochondrial mass, MDA-MB-231 cells were stained with nonyl acridine orange (NAO), a potential-independent dye that binds cardiolipin (Figure 1A). Metformin (Met) or a low (0.6 mM) concentration of 2DG achievable in vivo (0.6DG) had no effect on mitochondrial mass. The higher 2DG concentration (4.8 mM; referred to as 4.8DG), similar to glucose concentration in the medium (5.6 mM), increased mitochondrial mass to 130% of the control levels (not significant). The combined treatment with 5 mM metformin plus 0.6 mM 2 DG (Met + 0.6DG) increased the mitochondrial mass to about 165% of the control levels, while 5 mM metformin plus 4.8 mM 2DG (Met + 4.8DG) had no effect. Staining with Mitotracker Orange, which accumulates in the polarized mitochondria, also revealed a 125% increase in mitochondrial mass in 4.8DG and 150% in Met + 0.6DG treated cells (Figure 1B). A 769662, a direct AMPK activator, and rapamycin, an mTOR inhibitor, were used to test whether the AMPK activation or mTOR inhibition were sufficient to increase mitochondrial mass; no significant effect was found for either.

We next analyzed mitochondrial mass in BT-549 cells, another TNBC cell line displaying the detachment phenotype. Metformin and 2DG did not increase mitochondrial mass (NAO staining) in their standard medium (Figure S1A). However, when cells were adapted to the same medium as used for MDA-MB-231 cells (without pyruvate and insulin), similar trends were observed in both TNBC cell lines (Figure 1C).

There was no significant increase in mitochondrial mass in the medium without added glucose (containing only glucose from serum) in either TNBC cell line (Figure 1D, Figure S1B). Metformin treatment in the absence of added glucose had no additional effect in MDA-MB-231 cells, but significantly reduced mitochondrial mass in BT-549 cells.

In parallel, we determined the effects of metformin and 2DG on the total cell number and the percentage of dead cells. 4.8DG significantly reduced the total cell number (Figure 1E,F), with no increase in the number of dead cells (Figure S1C), indicating suppressed proliferation. The same was true for Met + 0.6DG in MDA-MB-231 cells, while its effect in BT-549 cells was even stronger. Met + 4.8DG reduced the total cell number even more (below the starting number), and significant cell death was observed in both cell lines (>30% Trypan Blue-positive, Figure S1C,D). Without added glucose, 2DG significantly decreased the MDA-MB-231 total cell number already at 0.6 mM (Figure 1G). PI-positive cells were observed for metformin in the absence of glucose (Figure S2), consistent with our previous study [30]. Overall, the increase in mitochondrial mass was observed with 4.8DG and Met + 0.6DG where cell proliferation was significantly suppressed but no direct effect on cell death was observed.

### 2.2. The Increased Mitochondrial Mass with Metformin and 0.6 mM 2DG Treatment Is Associated with Increased Mitochondrial Size in MDA-MB-231 Cells

**Figure 2:**
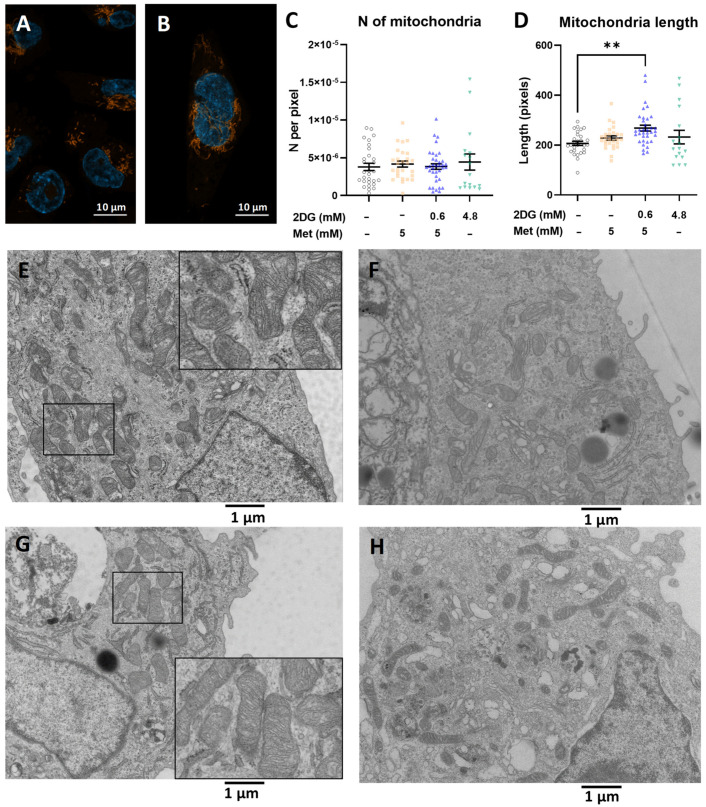
The effect of metformin and 2DG on mitochondrial morphology in MDA-MB-231 cells. MDA-MB-231 cells were treated with 5 mM metformin and/or 0.6 mM 2DG or 4.8 mM 2DG for 72 h with daily medium change. (**A**,**B**) Representative fluorescence micrographs were captured after Mitotracker Orange and Hoechst 33342 staining. (**C**–**H**) Representative transmission electron micrographs were captured for 2 to 3 independent experiments, and (**C**) the number of mitochondria per surface area of cytoplasm and (**D**) mean length of mitochondria were determined. Each data point represents data from individual micrographs and horizontal bars indicate mean ± SEM. (**E**–**H**) Representative micrographs of (**E**) control, (**F**) 5 mM metformin-, (**G**) 5 mM metformin + 0.6 mM 2DG- and (**H**) 4.8 mM 2DG-treated cells at 3900× magnification. Scale bars indicate (**A**,**B**) 10 μm or (**E**–**H**) 1 μm. ** *p* < 0.01 as determined by ANOVA.

As the increase in mitochondrial mass can result from either an increased number or size of mitochondria, we next evaluated the effects of metformin and 2DG on mitochondrial morphology and number. Fluorescence micrographs of Mitotracker Orange-stained MDA-MB-231 cells confirmed the increased mitochondrial mass per cell in Met + 0.6DG, but no clearly visible difference in the organization of the mitochondrial network was observed (Figure 2A,B). TEM micrographs revealed no observable effect on the number of mitochondria per unit area of cytoplasm (Figure 2C). To further support this result, we quantified the mitochondrial DNA. Indeed, none of the treatments resulted in changed mtDNA quantity (Figure S3). The Met + 0.6DG-treated cells had on average 30% longer mitochondria vs. control (Figure 2D,E–H), while Met or 4.8DG treatment showed a similar trend, with about a 15% increase in length (not significant). Apart from increased length, Met + 0.6DG did not induce apparent changes in mitochondrial morphology, such as cristae organization (Figure 2G). These results indicate that the increase in mitochondrial mass in the Met + 0.6DG treated cells is not associated with the production of novel mitochondria. but rather with a larger size of morphologically normal mitochondria.

**Figure 3:**
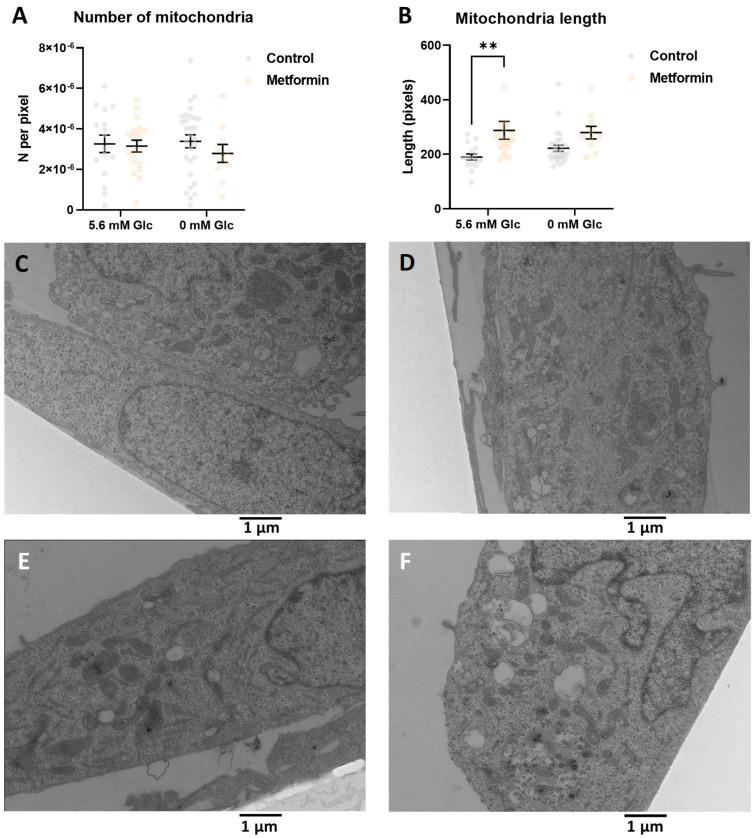
The effect of metformin on mitochondria in MDA-MB-231 cells as a function of glucose level. MDA-MB-231 cells were treated with 5 mM metformin in the RPMI medium supplemented with 5.6 mM or 0 mM glucose (Glc). The cells were treated for 48 h with daily medium change and TEM micrographs were captured. The (**A**) average number of mitochondria per surface area of cytoplasm and (**B**) mean length of mitochondria were determined. Each data point represents data from individual micrographs from two independent experiments and horizontal bars indicate mean ± SEM. ** *p* < 0.01 as determined by ANOVA. (**C**–**F**) Representative micrographs of the control with (**C**) 5.6 mM glucose, (**D**) 5 mM metformin 5.6 mM glucose, (**E**) control 0 mM glucose and (**F**) 5 mM metformin in 0 mM glucose at 3900× magnification. Scale bar = 1 μm.

To investigate the effect of glucose availability, we performed TEM analysis after 48 h due to cell death caused by metformin in the medium without added glucose after 72 h (Figure S2). Glucose itself did not affect the mitochondrial number or length. Metformin (Figure 3A–F) did not affect the number of mitochondria (Figure 3A), but a trend towards longer mitochondria was observed with metformin (not significant) in the medium without added glucose (Figure 3B). Overall, glucose availability seemed to have a limited impact on the mitochondria in MDA-MB-231 cells.

### 2.3. Seahorse Real Time ATP Production and Mito Stress Assay

**Figure 4:**
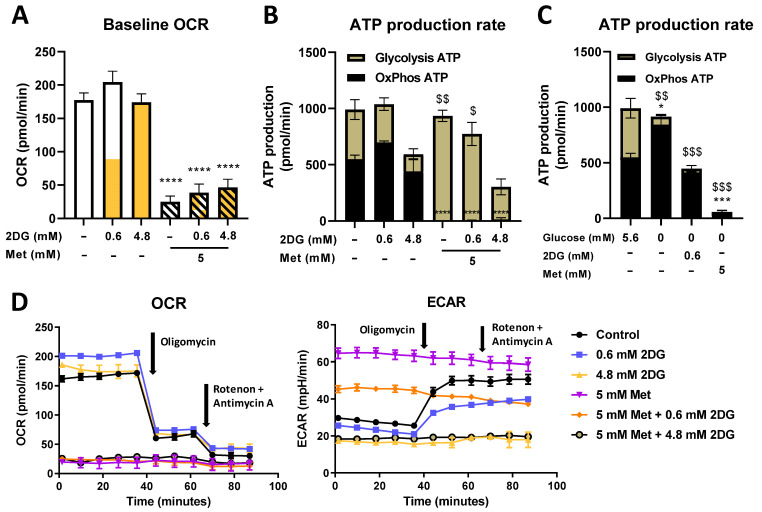
The effect of metformin and 2DG on ATP production from glycolysis and oxidative phosphorylation. MDA-MB-231 cells were treated for 48 h with the indicated compounds in RPMI medium supplemented with (**A**,**B**,**D**) 5.6 mM or (**C**) 0 mM glucose as indicated, with daily medium change. Following treatment, the oxygen consumption rate (OCR) and extracellular acidification rate (ECAR) were measured with Seahorse XFe24 analyzer using the Real Time ATP Production Assay. The results were corrected for relative cell number as determined by Hoechst staining. (**A**) Baseline OCR. (**B**,**C**) ATP production from oxidative phosphorylation and glycolysis was calculated according to the manufacturer’s instructions. (**D**) Representative OCR and ECAR timelines. The mean ± SEM for three independent experiments is shown. Data is color-coded according to treatment. **** *p* < 0.0001 as determined by ANOVA. For ATP production (**B**,**C**), * *p* < 0.05, *** *p* < 0.001, **** *p* < 0.0001 as determined by ANOVA for OxPhos ATP production; $ *p* < 0.05, $$ *p* < 0.01, $$$ *p* < 0.001 as determined by ANOVA for glycolytic ATP production.

To further understand how changes in mitochondrial mass in MDA-MB-231 cells were reflected in their mitochondrial function, baseline and maximal (after FCCP injection) oxygen consumption rate (OCR), we measured these values using the Seahorse Mito Stress Assay. Both Met and Met + 0.6DG treatment significantly decreased baseline and maximal OCR compared to the control (Figure 4A and Figure S4). 2DG did not significantly affect OCR. AMPK activator A 769662 had no observable effect on OCR levels.

We further measured ATP production from oxidative phosphorylation (OxPhos) and glycolysis using the Seahorse Real Time ATP Production Assay. In control cells, both processes contributed approximately half of the total ATP production (Figure 4B, representative OCR time-lapse in Figure 4D). 2DG did not significantly change OxPhos ATP production, though a trend toward higher OxPhos was observed for 0.6DG. Conversely, 2DG showed a dose-dependent trend towards lower glycolytic ATP production, reaching about 80% of the control levels with 0.62DG and 35% of the control levels with 4.8DG (not significant). All treatments with metformin completely suppressed OxPhos ATP production. With metformin alone, this was compensated with increased glycolysis, and so the total ATP production was unchanged. This compensation was incomplete in Met + 0.6DG- and absent in Met + 4.8DG-treated cells, so total ATP production was reduced to 75% and 30% of the control level, respectively.

In the medium without added glucose, glycolytic ATP production was completely suppressed as expected regardless of Met or DG treatment (Figure 4C). Untreated cells had increased OxPhos ATP production compared to the medium with 5.6 mM glucose (*p* < 0.05), and so total ATP production was unchanged. 0.6DG treatment reduced OxPhos ATP production back to control levels in 5.6 mM glucose. Total ATP production was reduced to about 50% of the control levels. Met treatment reduced OxPhos and total ATP production below 10% of the control levels. Overall, the low availability of glucose prevented the cells from compensating for the reduction in OxPhos with increased glycolysis, leading to lower total ATP production compared to 5.6 mM glucose.

### 2.4. Metformin and 2DG Treated MDA-MB-231 Cells Maintain Their Mitophagy

**Figure 5:**
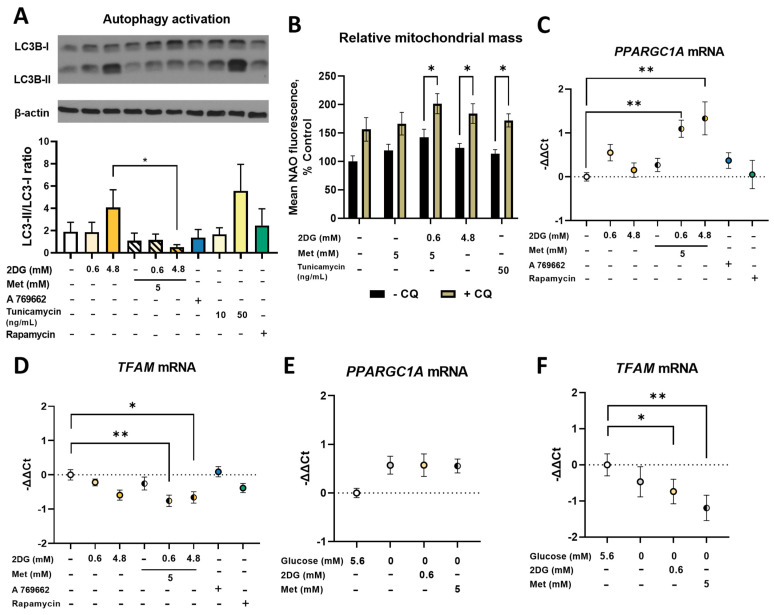
The effect of metformin and 2DG on mitochondrial biogenesis and autophagy. MDA-MB-231 cells were grown in the RPMI medium supplemented with (**A**–**D**) 5.6 mM or (**E**,**F**) 0 mM glucose and treated with indicated compounds for (**A**,**C**–**G**) 24 h or (**B**) 72 h. (**A**) The ratio of LC3-II to LC3-I was determined by Western blot. (**C**,**D**) Mean expression levels for (**C**,**E**) *PPARGC1A* mRNA and (**D**,**F**) *TFAM* mRNA were determined wit qRT-PCR. (**B**) MDA-MB-231 cells were treated with 4.8 mM 2DG or 5 mM metformin ± 0.6 mM 2DG in the presence or absence of 40 μM chloroquine (CQ) for 72 h with daily medium change and the mitochondrial mass determined with NAO staining. The mean ± SEM is shown for 3–4 independent experiments. Data is color-coded according to treatment (orange for 2DG, blue for A 769662, green for rapamycin, yellow for tunicamycin and hatching for metformin). * *p* < 0.05, ** *p* < 0.01 as determined by ANOVA. (Figure S11 Original western blot images).

The observed increase in mitochondrial mass in MDA-MB-231 cells could result from suppressed mitophagy. We therefore evaluated the overall activation of autophagy by the LC3-I to LC3-II conversion (Figure 5A). Untreated MDA-MB-231 cells expressed some LC3-II, indicative of some baseline autophagy activation. 4.8DG increased the LC3-II/LC3-I ratio to 2-fold higher than the control. On the other hand, we observed ~50% lower LC3-II/LC3-I ratios compared to the control or 2DG alone for Met, Met + 0.6DG and Met + 4.8DG treatments despite significant mTOR pathway suppression (Figure S5). Two-way ANOVA for metformin and 2DG showed this was significant only in Met + 4.8DG compared to 4.8DG. A 769662 or rapamycin did not have a marked effect on the LC3-II/LC3-I ratio. 2DG is known to inhibit protein glycosylation which could contribute to autophagy activation. We therefore also treated the cells with tunicamycin, a direct inhibitor of protein N-glycosylation [49] and a known endoplasmic reticulum (ER) stressor. Cells treated with tunicamycin indeed showed approximately 3-fold higher LC3-II/LC3-I ratio vs. control (Figure 5A), indicating that inhibited N-glycosylation was likely the main trigger of autophagy.

As decreased autophagy could indicate decreased mitophagy, we next quantified its contribution to the observed mitochondrial mass. The cells were treated with metformin and 2DG in the presence of chloroquine that inhibits autophagic processes by inhibiting lysosomal acidification, blocking mitophagy. Control cells treated with chloroquine had 150% increased mitochondrial mass vs. the untreated control (Figure 5B). This effect of chloroquine was preserved with Met + 0.6DG and 4.8DG, as chloroquine-treated cells had about 30 to 40% higher mitochondrial mass than their counterparts without chloroquine. Mitophagy was thus mostly preserved by Met + 0.6DG treatment, indicating that its inhibition was not a major contributor to increased mitochondrial mass.

### 2.5. Combined Metformin + 2-Deoxyglucose Treatment Induces Mitochondrial Biogenesis in MDA-MB-231

We next measured the induction of mitochondrial biogenesis by its master regulator *PPARGC1A* and its downstream effector *TFAM* using quantitative real-time PCR (qRT-PCR). As transcriptional changes precede the expression of proteins, we analyzed the mRNA levels after 24 h (Figure 5) of treatment. Both Met + 0.6DG and Met + 4.8DG increased *PPARGC1A* mRNA expression levels (−ΔΔCt ≈ 1) compared to the control (Figure 5C), while metformin or 2DG alone had no effect. The relative *TFAM* mRNA levels showed a dose-dependent trend lower with 2DG (−ΔΔCt ≈ 0.75 for 4.8DG, Figure 5D). While Met did not alter *TFAM* mRNA levels, we found lower levels with Met + 0.6DG and Met + 4.8DG vs. the control (−ΔΔCt ≈ –1). Similar but less pronounced effects were observed after 48 h treatment (Figure S6). Neither A 769662 nor rapamycin significantly altered *PPARGC1A* or *TFAM* mRNA levels.

In the medium without added glucose, we found a trend towards higher levels of *PPARGC1A* mRNA (−ΔΔCt ≈ 0.5, not significant, Figure 5E), while 0.6DG or Met treatment did not further increase them. *TFAM* mRNA levels were unaffected by glucose availability, but they were lowered by 0.6DG (−ΔΔCt ≈ −0.75) or Met (−ΔΔCt ≈ −1) treatment compared to control cells in 5.6 mM glucose (Figure 5F).

### 2.6. Metformin and 2DG Induce ER Stress by Suppressing N-Glycosylation in MDA-MB-231 Cells

**Figure 6:**
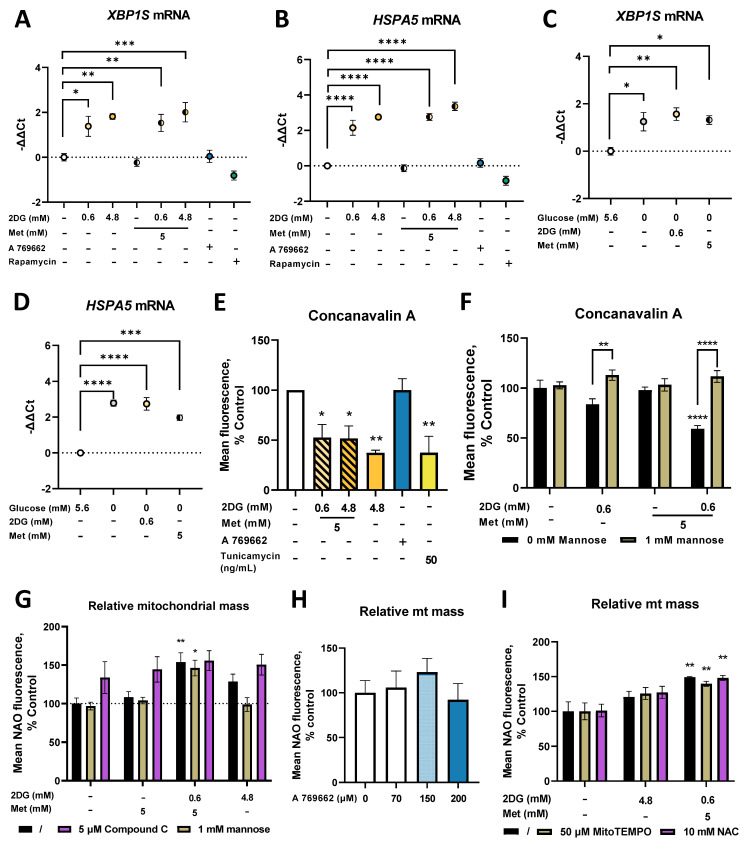
The effect of metformin and 2DG on ER stress markers and protein N-glycosylation in MDA-MB-231 cells. MDA-MB-231 cells were grown in the RPMI medium supplemented with (**A**,**B**,**E**–**H**) 5.6 mM or (**C**,**D**) 0 mM glucose and treated with indicated compounds for (**A**–**D**) 24 h, (**E**,**F**) 48 h or (**G**–**I**) 72 h. *XBP1S* (**A**,**C**) and *HSPA5* (**B**,**D**) mRNA levels were determined with qRT-PCR. Surface protein N-glycosylation was determined with Alexa 488-conjugated concanavalin A staining and flow cytometry (**E**,**F**). Mitochondrial mass was determined by NAO staining and flow cytometry (**G**–**I**). The means +/- SEM for (**E**–**F**,**H**,**I**) three, (**A**–**D**) four (**G**) or five independent experiments are shown. * *p* < 0.05, ** *p* < 0.01, *** *p* < 0.001, **** *p* < 0.0001 as determined by ANOVA.

In our study, mitochondrial mass was only increased in the presence of 2DG, which is known to inhibit N-glycosylation and induce ER stress [25,28,50]. We therefore next explored the effects of metformin and 2DG on these two processes. No changes in the relative mRNA levels of *XBP1*, a transcription factor induced by ER stress, were found (Figure S7). However, as only the isoform spliced by IRE1 in conditions of ER stress (*XBP1S*) serves as a transcription factor for the subsequent unfolded protein response (UPR) [51], we also measured this isoform specifically. Already after 24 h, 2DG significantly increased *XBP1S* mRNA levels vs. control (Figure 6A) with a significant effect already at 0.6 mM (−ΔΔCt ≈ 1.5 for 0.6DG; −ΔΔCt ≈ 2 for 4.8DG). An increase in *XBP1S* mRNA was also observed for both metformin + 2DG treatments (−ΔΔCt ≈ 2), while metformin alone had no effect. A 769662 and rapamycin had no effect on *XBP1S* mRNA. *XBP1S* mRNA levels were elevated (−ΔΔCt ≈ 1.2) in the medium without added glucose (Figure 6C), but Met or 2DG treatment alone did not further increase them (−ΔΔCt ≈ 1.5 vs. control at 5.6 mM glucose). Similar changes in *XBP1S* mRNA were also observed after 48 h, indicating a sustained transcriptional response (Figure S7). On the other hand, there were no changes in the unspliced isoform (*XBP1U*) thought to function as a negative feedback loop (Figure S7).

Both 0.6DG and 4.8DG significantly increased *HSPA5* (an ER chaperone induced as a result of UPR) mRNA vs. the control (−ΔΔCt ≈ 2 for 0.6DG, −ΔΔCt ≈ 3 for 4.8DG, Figure 6B). The same was true for Met + 0.6DG and Met + 4.8DG (−ΔΔCt ≈ 3), while Met had no effect. A 769662 and rapamycin also had no effect. As with *XBP1S*, *HSPA5* mRNA levels were significantly increased in the medium without added glucose (−ΔΔCt ≈ 3), and neither 0.6DG nor Met induced any further increase (Figure 6D). Similar trends were observed after 48 h (Figure S7). Overall, we found a robust transcriptional response in markers of ER stress and UPR in the cells treated with 2DG, both alone and in combination with metformin.

To confirm that the induced ER stress was linked to suppressed protein N-glycosylation, we stained the cells with concanavalin A, a lectin that specifically binds N-glycosylated proteins. Met + 0.6DG reduced the surface protein N-glycosylation (~40% of the control, Figure 6E). The effect of 4.8 mM 2DG was even stronger (~50% of the control) and similar to tunicamycin (~50% of the control). To confirm that this resulted from 2DG competition with other monosaccharides, we also performed the experiment in the presence of mannose, one of the main monosaccharides incorporated into N-linked glycans that can rescue glycosylation. While mannose itself did not affect protein glycosylation, it reversed the effect of 2DG and metformin + 2DG treatment (Figure 6F). This confirms the competitive inhibition of glycosylation by 2DG and its potentiation by metformin. Altogether, the results show that suppressed N-glycosylation due to 2DG or Met + 0.6DG leads to ER stress and UPR.

To explore the relation between N-glycosylation and mitochondrial mass, we next treated the cells with metformin and 2DG in the presence of mannose. While mannose did not block the increase in mitochondrial mass by Met + 0.6DG treatment, it reduced the levels of NAO to the control levels in 4.8DG treated cells (not significant, Figure 6G). This suggests that suppressed N-glycosylation or the subsequent ER stress or UPR likely play a role in increasing mitochondrial mass, but this is not necessary in Met + 0.6DG treated cells.

Both metformin, 2DG and especially their combination are known to activate AMPK [30], which can induce mitochondrial biogenesis [13]. We confirmed AMPK activation for 4.8DG and especially Met + 0.6DG by measuring the phosphorylation of acetyl-CoA carboxylase (ACC), the downstream target of AMPK (Figure S7). However, compound C, an AMPK inhibitor, did not prevent the increase in mitochondrial mass with Metf+0.6DG or 4.8DG (Figure 6G). Still, compound C did not fully reduce ACC phosphorylation (Figure S7), and a trend towards increased mitochondrial mass was observed in cells treated with 150 µM A 769662 concentration (Figure 6H) where increased ACC phosphorylation was observed (Figure S7). AMPK activation therefore likely plays a role in increasing mitochondrial mass in metformin and 2DG treatment.

As both electron transfer chain inhibition and ER stress can lead to increased ROS production, we finally treated the cells with metformin and 2DG in the presence of antioxidants. Neither mitochondria-specific (MitoTEMPO) nor unspecific (N-acetylcysteine, NAC) antioxidants were able to block the increase in mitochondrial mass (Figure 6I), indicating that ROS generation is unlikely to be the main trigger for increased mitochondrial mass.

### 2.7. Combined Treatment with Metformin and 2DG Decreases PD-L1 Expression in MDA-MB-231 Cells

**Figure 7:**
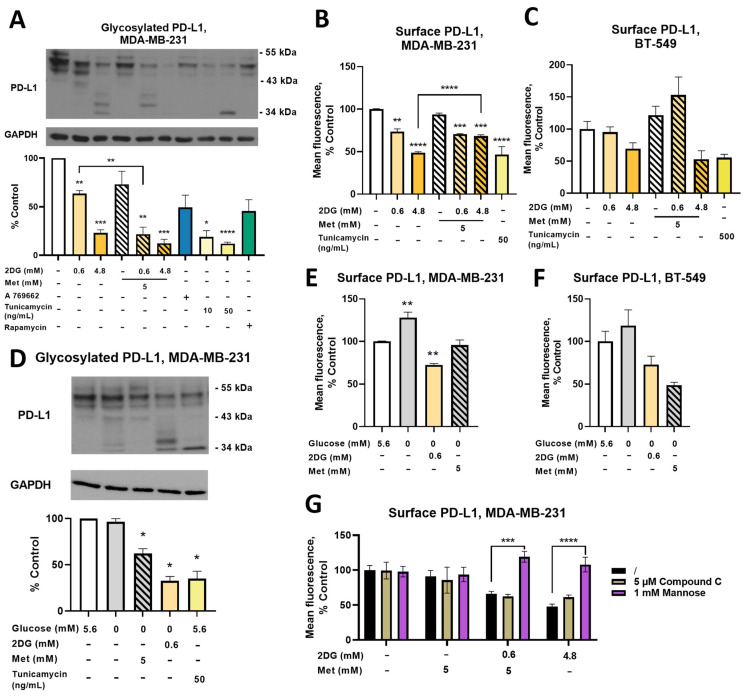
The effect of metformin and 2DG on PD-L1 expression in TNBC cells. MDA-MB-231 and BT-549 cells were grown in the RPMI medium supplemented with (**A**–**C**,**G**) 5.6 mM or (**D**–**F**) 0 mM glucose and treated with indicated compounds for (**A,D**) 24 h or (**B**,**C**, **E**–**G**) 48 h with daily medium change. (**A**,**D**) PD-L1 expression was investigated with Western blot and bands in the 43 kDa–55 kDa range representing normally glycosylated PD-L1 were quantified by densitometry. (**B**,**C**,**E**–**G**) Surface PD-L1 expression was determined by flow cytometry. (**G**) MDA-MB-231 cells were treated with metformin and/or 2DG in the presence or absence of 5 μM compound C or 1 mM mannose and the surface PD-L1 expression determined with flow cytometry. Mean ± SEM is shown for three independent experiments. Data is color-coded according to treatment (orange for 2DG, blue for A 769662, green for rapamycin and hatching for metformin; gray indicates low glucose). * *p* < 0.05, ** *p* < 0.01, *** *p* < 0.001, **** *p* < 0.0001 as determined by ANOVA. (Figure S11 Original western blot images).

Metformin and 2DG have multiple actions, among them deglycosylation and ER degradation of the immune-checkpoint protein programmed death-ligand 1 (PD-L1) [42,43]. We therefore analyzed how metformin- and 2DG-induced protein deglycosylation and ER stress will affect PD-L1 expression and glycosylation in MDA-MB-231 cells. 2DG induced a dose-dependent decrease in normally glycosylated (43 kDa–55 kDa) PD-L1 (to ~60% of the control for 0.6DG and ~20% of the control for 4.8DG, Figure 7A). Met alone decreased PD-L1 to ~70% of the control (not significant), while both metformin + 2DG treatments decreased glycosylated PD-L1 levels to ~20% of the control for Met + 0.6DG. While a significant interaction of metformin and 2DG on PD-L1 could not be confirmed, the effect of Met + 0.6DG was significantly stronger than 0.6DG (Figure 7A). Bands at lower molecular weight (~34 kDa) absent in the control were observed with 4.8DG and Met + 0.6DG, indicating PD-L1 deglycosylation. Glycosylated PD-L1 levels were not significantly altered by A 769662 or rapamycin. Tunicamycin decreased glycosylated PD-L1 already at 10 ng/mL, with 50 ng/mL further decreasing it to about 10% of the control. PD-L1 bands at ~34 kDa confirming deglycosylation were also observed. Taken together, 2DG decreased glycosylated PD-L1 already at 0.6 mM 2DG, and its effect was potentiated by concurrent metformin treatment. Direct inhibition of protein N-glycosylation seemed to be the most important, while AMPK activation did not appear to play a major part.

We next measured surface PD-L1 relevant for its function. After 24 h, 2DG reduced surface PD-L1 in a dose-dependent manner to ~90% of the control for 0.6DG and ~70% of the control for 4.8DG (Figure S8). The effect of tunicamycin was even stronger (~60% of the control). Metformin only affected PD-L1 in combination with 4.8 mM 2DG (~85% of the control for Met + 4.8DG). These effects were much more pronounced after 48 h (Figure 7B). 0.6DG reduced surface PD-L1 levels to 75%, while 4.8DG reduced them to 50% of the control. While Met did not impact surface PD-L1, both Met + 0.6DG and Met + 4.8DG led to a ~70% decrease. Two-way ANOVA revealed an interaction between 2DG and metformin, as 2DG did not have a linear dose-dependent effect in the presence of metformin and surface PD-L1 was higher in Met + 4.8DG compared to 4.8DG (Figure 7B). Overall, the reduced PD-L1 glycosylation with 2DG and Met + 0.6DG was also reflected in the decreased surface PD-L1 expression.

In BT-549 TNBC cells, surface PD-L1 levels were decreased by both 4.8DG (~65% control, not significant) and Met + 4.8DG (~50% control, not significant, Figure 7C). The effect of Met + 0.6DG was, however, markedly different as it increased surface PD-L1 by ~50% above the control (not significant). The effect of tunicamycin was preserved (~50% control, not significant), but required a higher concentration (500 ng/mL) compared to MDA-MB-231 cells. Thus, the effect of glycosylation inhibitors (2DG and tunicamycin) on reduced PD-L1 expression was maintained across TNBC cell lines, but the potentiating effect of metformin was absent in BT-549 cells.

Exploring the effect of glucose availability in MDA-MB-231 cells, total normally glycosylated PD-L1 levels were unchanged in the medium without added glucose, although some very weak bands were observed at ~34 kDa (Figure 7D). However, surface PD-L1 levels were higher at both 24 h and 48 h (Figure 7E, Figure S8). Both 2DG and metformin in the absence of added glucose reduced total normally glycosylated PD-L1 (~60% and ~30% of the control, respectively), although only 2DG had visible bands at ~34 kDa. Both treatments prevented the increase in surface PD-L1 levels in the absence of added glucose, with 2DG even significantly reducing them after 48 h. Similar trends were observed in the medium without added glucose in BT-549 cells (Figure 7F), increasing surface PD-L1 (not significant), while metformin and 2DG both reduced surface PD-L1 expression (not significant). Overall, the very low glucose concentration led to increased surface PD-L1 expression in both TNBC cell lines which was normalized by 0.6DG or Met treatment.

As established by previous research on metformin and 2DG, PD-L1 expression can be reduced by phosphorylation by AMPK or direct protein deglycosylation. We therefore treated MDA-MB-231 cells with metformin and 2DG in the presence of either 1 mM mannose or 5 μM compound C (6-[4-(2-Piperidin-1-ylethoxy) phenyl]-3-pyridin-4-ylpyrazolo [1,5-a]pyrimidine), a direct AMPK inhibitor (Figure 7G). Compound C had no major effect on surface PD-L1, demonstrating that AMPK activation was not crucial for PD-L1 reduction. On the other hand, mannose completely blunted the effect of both 4.8DG and Met + 0.6DG, increasing PD-L1 levels back to control levels. These results demonstrate that suppressed protein N-glycosylation of PD-L1 rather than AMPK activation is the main mechanism responsible for lower PD-L1 expression.

### 2.8. Metformin and 2DG Do Not Increase Mitochondrial Mass in Jurkat Cells

**Figure 8:**
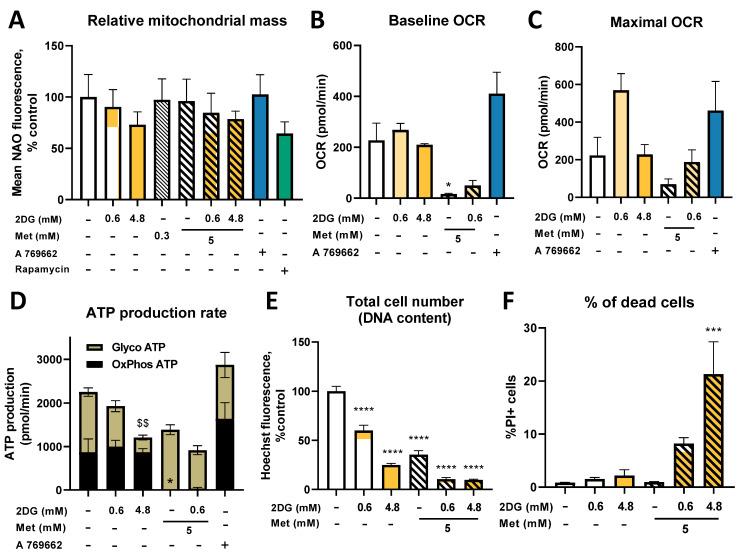
The effect of metformin and 2DG on Jurkat cell mitochondria. Jurkat cells were treated with 5 mM metformin and/or 0.6 mM 2DG or 4.8 mM 2DG for (**A**,**E**,**F**) 72 h or (**B**–**D**) 48 h. (**A**) Relative mitochondrial mass was determined with NAO staining. (**B**–**D**) Baseline and maximal OCR was determined using Seahorse Mito Stress Test and the ATP production calculated according to the manufacturer’s instructions. (**E**) Total cell number was determined by measuring DNA content using Hoechst 33342 staining. (**F**) The percentage of dead cells was determined by PI staining and flow cytometry. Mean ± SEM is shown for three independent experiments. Data is color-coded according to treatment (orange for 2DG, blue for A 769662, green for rapamycin and hatching for metformin). * *p* < 0.05, *** *p* < 0.001, **** *p* < 0.0001 as determined by ANOVA. For ATP production, * denotes *p* < 0.05 for OxPhos and $$ *p* < 0.01 for glycolytic ATP as determined by ANOVA.

The reduction in surface PD-L1 levels in TNBC cells and other published reports [40,41,42,43] suggest that 2DG and metformin could partially mediate their anti-cancer effect by modulating the anti-tumor immune response. We therefore examined the effects of 2DG and metformin on Jurkat cells as a model for T cells. We first measured the effect of metformin and 2DG on mitochondrial mass, which is closely linked to in vivo function of tumor-infiltrating T cells [52]. Metformin, 2DG or their combination did not significantly increase mitochondrial mass (Figure 8A). Glucose availability also did not influence the effect of metformin on mitochondrial mass in Jurkat cells (Figure S9). Both 2DG and metformin increased autophagy (4 -fold increase for 2DG, 2.5-fold increase for metformin) in Jurkat cells (not significant, Figure S9), which could play a role in lowering mitochondrial mass.

We next explored the effect of metformin and 2DG on baseline and maximal OCR. 0.6DG slightly increased (~15%) baseline and substantially increased (~300% of the control) maximal OCR (not significant, Figure 8B,C), while 4.82DG had no effect on either. Met and Met + 0.6DG (not significant) almost completely suppressed baseline OCR. Maximal OCR showed a trend lower only in metformin. Jurkat cells had a very glycolytic phenotype, producing ~70% of ATP by glycolysis (Figure 8D). 2DG showed a dose–dependent trend towards reduced glycolytic ATP production with a small compensatory increase in OxPhos. Met and Met + 0.6DG completely suppressed oxidative ATP but did not influence glycolytic ATP production, reducing the total ATP production to ~75% of the control (not significant). A 769662 showed a trend towards higher baseline and maximal OCR, as well as OxPhos ATP production (not significant). Overall, in contrast to MDA-MB-231 cells, Jurkat cells had almost no spare capacity in either OxPhos or glycolysis, so both 2DG and metformin treatment reduced the total ATP production.

Due to the restricted energy status, we also determined the effect of metformin and 2DG on Jurkat cell number and cell death. 2DG reduced Jurkat cell number in a dose-dependent manner (60% and 25% of the control for 0.6DG and 4.8DG, respectively, Figure 8E). Metformin reduced the cell number to about 35% of the control, while both Met + 0.6DG and Met + 4.8DG reduced it to 10%. The percentage of dead cells was, however, only increased by Met + 4.8DG, although a similar trend was observed in Met + 0.6DG (Figure 8F). The inability of Jurkat cells to compensate for ATP production with metformin or 2DG treatment was therefore also reflected in their reduced cell number.

### 2.9. Combined Treatment with Metformin and 2DG Suppresses PD-L1 and PD-1 Expression in Jurkat Cells

**Figure 9:**
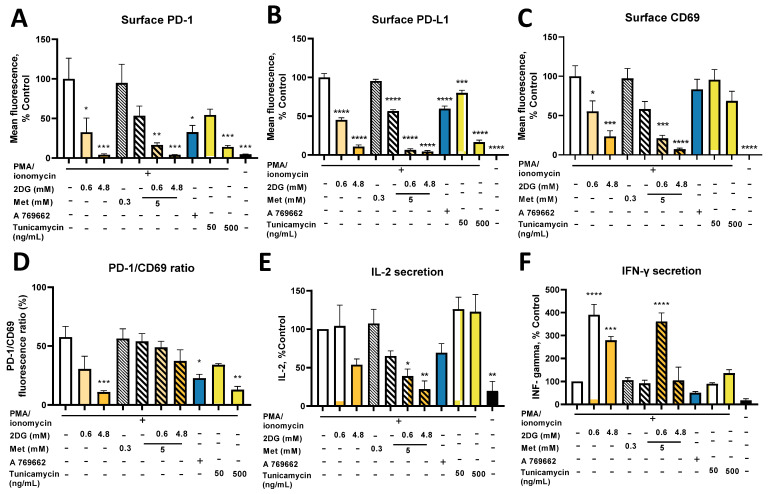
The effect of metformin and 2DG on PD-L1/PD-1 axis and effector functions in Jurkat cells. Jurkat cells were activated with 1.0 μM ionomycin and 25 ng/mL PMA and treated with metformin and/or 2DG for 24 h. (**A**) Surface PD-1, (**B**) PD-L1 and (**C**) CD69 expression were determined using flow cytometry. The ratio of PD-1 and CD69 fluorescence was calculated and normalized to the control (**D**). IL-2 and IFN-γ secretion was measured with ELISA (**E**,**F**). Mean ± SEM is shown for three independent experiments. Data is color-coded according to treatment (orange for 2DG, blue for A 769662, green for rapamycin, yellow for tunicamycin and hatching for metformin). * *p* < 0.05, ** *p* < 0.01, *** *p* < 0.001, **** *p* < 0.0001 as determined by ANOVA.

PD-1 is the ligand for PD-L1 expressed on activated T cells. As Jurkat cells do not express PD-1 without activation, we activated them with PMA and ionomycin concurrent with metformin and 2DG treatment (Figure 9A). 2DG strongly suppressed PD-1 expression to ~25% of the control at 0.6 mM and 5% of the control at 4.8 mM; the same effect of 2DG was observed in the presence of metformin (15% of the control for Met + 0.6DG). Met reduced PD-1 levels to ~50% of the control (not significant). A 769662 also reduced PD-1 levels to ~35% of the control. The effect of tunicamycin was dose-dependent, reaching ~20% of the control at 500 ng/mL. PD-1 expression was therefore reduced by both AMPK activators and glycosylation inhibitors, so both mechanisms could be involved in the effect of metformin and 2DG on lower PD-1 levels.

Since Jurkat cells are themselves of malignant origin and PD-L1 overexpression could help them to avoid the immune system in vivo, we also measured PD-L1 expression. Jurkat cells expressed almost no PD-L1 in the basal state, while activation with PMA/ionomycin induced robust PD-L1 expression (Figure 9B). 2DG treatment reduced surface PD-L1 in a dose-dependent manner (~50% of the control for 0.6DG; ~10% of the control for 4.8DG). Met only reduced PD-L1 to ~60% of the control, while Met + 0.6DG and Met + 4.8DG suppressed PD-L1 to under 10% of the control. A 769662 reduced PD-L1 to 60% of the control. Tunicamycin already had a significant effect at 50 ng/mL, reaching ~15% of the control at 500 ng/mL. PD-L1 expression was also significantly reduced to ~50% in the medium without added glucose (Figure S10). Furthermore, 0.6DG or Met at 0 mM glucose further suppressed PD-L1 to under ~10% of the control. As for PD-1, both metformin and 2DG reduced surface PD-L1, and both AMPK and glycosylation inhibition could be involved.

Both glycolytic and mitochondrial metabolism are involved in T cell activation and their inhibition could block Jurkat cell activation, especially as both metformin and 2DG suppressed the mTOR pathway [53] (Figure S10A). We therefore measured the expression of CD69, an early marker of T cell activation (Figure 9C). Both drugs significantly reduced CD69 expression in activated Jurkat cells in a dose-dependent manner, with Met + 4.8DG having the strongest effect. Conversely, A 769662 and tunicamycin only had a mild effect (not significant). To estimate how much this contributed to reduced PD-1 expression, we calculated the ratio of relative PD-1 and CD69 fluorescence (Figure 9D). We found that 4.8DG, A 769662 and 500 ng/mL tunicamycin significantly reduced the PD-1/CD69 ratio, so the effect of 2DG but not metformin was stronger on PD-1 than activation, and likely caused by decreased N-glycosylation.

To examine the Jurkat cell effector functions, we measured IL-2 and IFN-γ secretion after activation (Figure 9E,F, absolute concentrations in Figure S10). 4.8DG and Met expectedly lowered IL-2 levels (~50% of the control, not significant) and Met + 0.6DG and Met + 4.8DG treatment showed an even stronger reduction in IL-2 levels (not significant). The AMPK activator A 769662 also reduced IL-2 levels to ~50% of the control (not significant), while tunicamycin had no observable effect. While we observed no effect of metformin on IFN-γ secretion, it was surprisingly increased to about 350% of the control levels by 0.6DG and Met + 0.6DG. A similar effect was noted for 4.8DG (~275% of the control) but not tunicamycin or A 769662. In the medium without added glucose (Figure S10B), IL-2 levels were ~60% of those at 5.6 mM glucose (not significant) and there was a further trend lower by Met or 0.6DG treatment (not significant). Glucose availability did not influence IFN-γ secretion, as we still observed the same increase with 0.6DG without added glucose, while metformin had no effect (Figure S10C). Overall, while metformin and/or 2DG reduced IL-2 secretion, the effect of 0.6DG on IL-2 was less pronounced than that on PD-1 expression, while IFN-γ secretion was preserved or even increased by metformin and 2DG treatment. This indicates at least partially preserved effector functions, suggesting that the suppressive effect of metformin and 2DG on PD-1/PD-L1 axis is stronger that than on Jurkat cell effector functions.

## 3. Discussion

Mitochondria play a crucial role in cancer proliferation and anchorage-independent growth during the process of metastasis formation. Metformin and 2DG are two metabolic drugs being investigated in the context of cancer prevention and treatment. However, due to their pleiotropic actions, their mechanisms of anticancer effect are still being studied. In the present study, we explored the effect of metformin, 2DG and their combination on the mitochondrial mass and biogenesis in triple-negative breast cancer (TNBC) cells in vitro. Increased mitochondrial mass and related adaptive processes could help some of the cancer cells and cancer stem cells to survive the anticancer treatment with metformin and 2DG. Importantly, mitochondrial functions in general were shown to be crucial for the survival of detached cancer cells [16,17].

As the main in vitro model, we therefore used two TNBC cell lines (MDA-MB-231 and BT-549) resistant to anoikis for which we have observed the phenomenon of detachment and survival in anchorage-independent conditions after treatment with metformin + 0.6 mM 2DG (Met + 0.6DG) or 4.8 mM 2DG (4.8DG) [16,30,54]. This could be especially relevant in the nutrient-poor tumor microenvironment, so we also analyzed the effects of metformin and 2DG in the absence of glucose. Additionally, as cancer cells in the tumor microenvironment compete for nutrients with infiltrating T cells [48], we also studied the effects of metformin and 2DG on Jurkat cells as a simple T cell model. It has recently been shown that both metformin and 2DG can partially abrogate the ability of cancer cells to suppress the functionality of T cells through the PD-1/PD-L1 axis, and that metformin can partially improve response to immune checkpoint inhibitors (ICI) in cancer therapy [42,43]. We therefore also evaluated the effect of metformin and 2DG on PD-L1 and PD-1 expression on TNBC and Jurkat cells, respectively.

### 3.1. Metformin and 2DG Induce Mitochondria Enlargement in TNBC Cells

We found that the combination of metformin and the low (0.6 mM) concentration of 2DG achievable in vivo (Met + 0.6DG) increased mitochondrial mass in MDA-MB-231 and BT-549 TNBC cells (Figure 1). This increase was associated with increased length rather than number of mitochondria, suggesting either the enlargement of existing mitochondria or increased mitochondrial fusion of newly formed mitochondria. Despite this, there was no obvious change in the overall organization of the mitochondrial network or the structure of the cristae. Although the respiratory function of mitochondria in metformin plus 2DG-treated cells was completely suppressed due to the effect of metformin itself, we can speculate that other mitochondrial functions are more or less preserved. Increased mitochondrial mass could thus provide MDA-MB-231 cells with a survival advantage, especially in anchorage-independent conditions [16,17,32,55].

Our results demonstrate that despite the initially suppressed autophagy activation, mitophagy was preserved with Met + 0.6DG treatment. Therefore, the observed increase in mitochondrial mass was a result of increased mitochondrial biogenesis (in terms of forming additional membranes and matrix space) and not due to suppressed mitophagy. This was also supported by increased mRNA levels of *PPARGC1A*, the gene for PGC-1α, the master regulator of mitochondrial biogenesis that can also increase mitophagy in TNBC cells [56]. Contrary to our expectation, we found the opposite trend in mRNA levels of *TFAM*, its downstream effector. This observation was consistent with no increase in the mitochondrial DNA copy number, as TFAM is involved in mitochondrial DNA replication [57], but is not always a good marker of mitochondrial biogenesis [58]. This is also consistent with no change in the number of mitochondria. Overall, the results indicate that the increase in mitochondrial mass is associated with mitochondrial biogenesis in terms of increased size and forming new mitochondrial mass, but not new mitochondria per se. While the two processes are usually linked, they are still incompletely understood and further advances in the field will be necessary in the future [59,60].

We found a similar but less pronounced effect on mitochondrial mass for 4.8 mM 2DG (4.8DG). We could not confirm any changes in the number or size of mitochondria, likely due to the small effect of 4.8DG on mitochondrial mass. Though the two treatments behaved similarly in many parameters including maintained mitophagy, we found no increase in *PPARGC1A* mRNA for 4.8DG, so its effects on mitochondrial biogenesis are less clear.

### 3.2. The Role of Energy Stress and Endoplasmic Reticulum Stress in Inducing Mitochondrial Biogenesis in MDA-MB-231 Cells

As metformin and 2DG are metabolic drugs that block ATP generation, we expected the TNBC cells to increase their mitochondrial mass primarily in response to energy stress (reduced ATP production) to potentially increase their respiratory capacity. Our results confirm this, as we saw no increase in mitochondrial mass in 0.6DG or metformin alone where total ATP production was unchanged. On the other hand, total ATP production was decreased by 30–40% in Met + 0.6DG and 4.8DG where increased mitochondrial mass and size were observed. This is consistent with a previous study showing mitochondria elongation or fusion under starvation or other conditions of increased energy needs [61,62,63,64,65]. However, mitochondrial mass was unchanged in Met + 4.8DG where total ATP production was severely restricted. Therefore, mitochondrial biogenesis seems to require both sufficient energy stress and an adequate energy supply.

AMPK is the canonical sensor of energy stress and a major activator of PGC-1α, thus being an important trigger of mitochondrial biogenesis in several cell types [12,13]. We have previously found that while metformin alone did not strongly activate AMPK, the combination of metformin and 2DG resulted in very strong activation in MDA-MB-231 cells [30]. Strong activation (as measured by ACC phosphorylation) was also observed for 4.8DG (Figure S7). AMPK activation could therefore be a possible mediator of the effect on Met + 0.6DG. However, we were unable to fully recapitulate its effect with AMPK activator A769662, achieving only about a 20% increase over baseline that was not significant even at 150 μM (Figure 6H). Furthermore, AMPK inhibitor compound C did not block the increase in mitochondrial mass (Figure 6G). However, these results could be explained by the incomplete activation or inhibition of AMPK by A 769662 and compound C, respectively, as evidenced by ACC phosphorylation (Figure S7). Nevertheless, they also suggest the possibility that AMPK activation is not the sole or main trigger of mitochondrial enlargement in Met + 0.6DG treated cells. We also showed that mTOR pathway inhibition is not crucial for this, as rapamycin treatment did not affect mitochondrial mass (Figure 1) or *PPARGC1A* mRNA expression. All of this indicates the possibility that another effect of metformin and 2DG distinct from energy stress or the energy sensing signaling pathways could potentially be involved.

We only observed marked increases in mitochondrial mass when 2DG was present, and these were not fully recapitulated by the absence of glucose. This has lead us to shift our attention to the effects of 2DG distinct from glycolysis inhibition. Importantly, we have observed consistent effects of 2DG on reduced protein N-glycosylation that were recapitulated by tunicamycin and rescued by mannose, consistent with the published literature [25,28,35,36,55]. The effect of 2DG on N-glycosylation was potentiated by concurrent metformin treatment, as has previously been observed [23]. We found consistent effects of both 0.6 mM and 4.8 mM 2DG on significantly increased spliced form of *XBP1* (*XBP1S*) and *HSPA5* mRNA levels, both alone and in combination with metformin (Figure 6). This indicates the presence of substantial endoplasmic reticulum (ER) stress and a robust activation of unfolded protein response (UPR), consistent with the published literature for 2DG [25,28,29,35,36] and observed with the inhibition of the hexosamine pathway [66].

**Figure 10:**
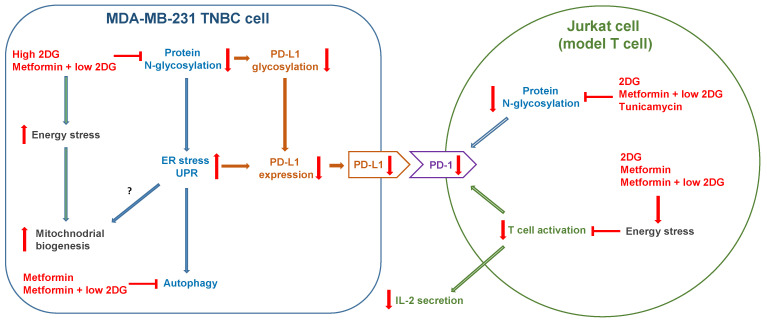
Overview of the effects of metformin and 2DG on mitochondria and PD-L1/PD-1 axis in MDA-MB-231 and Jurkat cells. High (4.8 mM) 2DG and metformin plus low (0.6 mM) 2DG treatments induce energy stress leading to increased mitochondrial biogenesis in MDA-MB-231 cells. High 2DG also inhibits protein N-glycosylation, leading to ER stress. Metformin potentiates the effect of 2DG, leading to the same effect with combined metformin plus low 2DG treatment. The increased ER stress seems to also play a role in increased mitochondrial biogenesis. Inhibited protein N-glycosylation leads to decreased PD-L1 glycosylation and surface expression. In activated Jurkat cells, the same inhibitory effect of metformin and 2DG on protein N-glycosylation decreases surface PD-1 expression. 2DG, metformin and metformin plus low 2DG also induce energy stress, blocking not only PD-1 expression, but also Jurkat cell activation and IL-2 secretion.

The elevated ER stress and UPR thus seemed to be related to increased mitochondrial mass for 4.8DG and Met + 0.6DG, where both the increase in mitochondrial mass as well as markers of ER stress were observed. This suggests the increased mitochondrial biogenesis in MDA-MB-231 cells could partially result from the activation of ER stress, which was shown to be able to induce mitochondrial biogenesis [55,67]. However, the levels of ER stress markers were very similar between 0.6DG and Met + 0.6DG, despite very different effects on mitochondrial mass. Furthermore, tunicamycin, a well-known ER stressor [68], only slightly increased mitochondrial mass (Figure 5B). Taken together, this indicates that while suppressed protein N-glycosylation and ER stress could play a role in increasing mitochondrial mass, they are not sufficient, and some degree of energy stress is also required (Figure 9). It is important to note that 2DG-induced ER stress can activate AMPK through Ca^2+^/calmodulin-dependent kinase kinase-β (CaMKKβ) [36]. This could account for a part of AMPK activation induced by Met + 0.6DG and especially 4.8DG, where mannose was able to prevent the increase in mitochondrial mass. Overall, both the energy and ER stress induced by metformin and 2DG seem to be important for increased mitochondrial biogenesis (Figure 10), since neither of them are sufficient for increased mitochondrial mass.

ER stress was shown to induce ROS generation, which can also be generated directly by ETC inhibition. These ROS, as well as PERK (one of the UPR kinases) directly, can activate the oxidative stress response transcription factor NRF2 which is also involved in mitochondrial biogenesis [69]. Therefore, ROS could play a role in mitochondrial biogenesis in TNBC cells. However, we have not observed any effects of antioxidants on increased mitochondrial mass (Figure 6I), indicating that ROS were not the main trigger of mitochondrial biogenesis.

We should also note that 4.8DG and Met + 0.6DG significantly reduced the MDA-MB-231 total cell number (Figure 1), with no major increase in cell death (Figure S2), which indicates suppressed proliferation. However, a major increase in cell death was observed for Met + 4.8DG (Figure S3). The increase in mitochondrial mass therefore seems to correlate with suppressed proliferation but not cell death. We and others have shown previously that metformin + 0.6 mM 2DG caused an arrest in the M and G2 phases in cancer cells [22,30], while tunicamycin (which does not substantially increase mitochondrial mass) was shown to induce cell arrest in G0/G1 phase [70]. We therefore speculate that the observed differences in mitochondrial biogenesis are partially due to different effects on proliferation. The regulation of mitochondrial biogenesis was shown to be linked to the cell cycle [71,72]. For example, cyclin D1 inhibited mitochondrial biogenesis in hepatocytes and its deficiency increased both mitochondrial mass and length [73]. Metformin and its combination with 2DG are known to reduce cyclin D1 expression in TNBC cells [74,75] and endothelial cells [23], respectively. It is therefore possible that these alterations in cyclin D1 levels are involved in increasing mitochondrial mass with metformin and 2DG treatment in TNBC cells.

### 3.3. Metformin and 2DG Do Not Increase Mitochondrial Mass in Jurkat Cells

As mitochondrial mass is also a key determinant of the metabolic fitness of tumor-infiltrating T cells which governs their ability to mount an effective immune response [48], we investigated the effect of metformin and 2DG on mitochondria in Jurkat cells. We found no increase in mitochondrial mass with Met treatment, while 4.8DG and Met + 0.6DG actually decreased mitochondrial mass (Figure 1). On the other hand, Jurkat cells had a very low level of constitutive autophagy activation which was slightly (not significant) increased by both metformin and 2DG treatment. This could potentially help to explain the decreased mitochondrial mass in 4.8DG, Met + 0.6DG and Met + 4.8DG treated Jurkat cells. Nevertheless, the low magnitude of autophagy activation (especially compared to rapamycin) indicates that additional mechanisms are likely involved. Jurkat cells were in general also more susceptible to metformin treatment or glucose starvation compared to MDA-MB-231 cells (Figure S9), with a significantly decreased cell number with metformin or 2DG treatment (Figure 8E,F).

### 3.4. The Effect of Metformin and 2DG on PD-L1/PD-1 Axis through Suppressed Protein N-Glycosylation

In addition to the relative metabolic capacity of tumor cells and infiltrating T cells, one of the most important factors influencing the anti-tumor immune response in general is their interaction through the PD-1/PD-L1 axis. Upregulated PD-L1 expression allows cancer cells to inhibit T cell effector functions, enabling the immune escape of the tumor. Moreover, the PD-L1/PD-1 interaction has also been shown to boost glycolysis in cancer cells while inhibiting it in T cells, providing cancer cells with a metabolic advantage in the nutrient-poor tumor microenvironment [48]. It is therefore crucial to find additional strategies to inhibit the PD-1/PD-L1 axis. It has recently been shown that metformin and 2DG both affect PD-L1 expression on TNBC cells by inducing an AMPK dependent degradation of PD-L1 in the ER and deglycosylation, respectively [42,43]. The synergistic effect of metformin and 2DG on protein N-glycosylation and ER stress observed in the published literature [23] and the present study leads us to study the effects of metformin and 2DG on PD-L1 and PD-1 expression in TNBC and Jurkat cells.

Our results show that 2DG reduced glycosylated PD-L1 levels in a dose-dependent manner (Figure 7A) in MDA-MB-231 cells. As PD-L1 glycosylation is required for its function [76,77], reducing PD-L1 glycosylation with metformin and 2DG could further improve anti-tumor immune responses. While we did not observe a significant decrease in glycosylated PD-L1 with metformin alone, there was major suppression by metformin + 2DG. This could indicate a possible synergism between metformin and 2DG, which we could not confirm with two-way ANOVA. Additionally, these changes were also reflected in reduced surface PD-L1 levels (Figure 7C,D), further supporting a potential beneficial effect of metformin and 2DG in anti-tumor immunity. In BT-549 cells, the effect of 2DG and tunicamycin on decreased PD-L1 expression was preserved, while the metformin and metformin + 0.6 mM 2DG treatments actually led to increased surface PD-L1. These results indicate that the effect of glycosylation inhibition by 2DG on decreased PD-L1 levels is consistent across the two TNBC lines. However, there is considerable variation between cell lines in response to metformin.

Interestingly, glucose deprivation did not lower glycosylated PD-L1 (Figure 7B), and surface PD-L1 levels were actually higher in the medium without added glucose in both TNBC lines. A similar finding was recently reported for renal carcinoma where low glucose also increased PD-L1 expression [78]. This indicates that under glucose-deprived conditions or energy stress, PD-L1 expression on TNBC cells can be increased. Such an increase in metabolic stress conditions could potentially also explain the increase in PD-L1 expression with Met + 0.6DG in BT-549 cells. Importantly, metformin or 0.6 mM 2DG in low-glucose conditions normalized or even reduced PD-L1 levels (Figure 7D). As tumor cells and infiltrating T cells face a nutrient-poor environment in vivo, this observation could be important for the use of metformin or 2DG as an adjuvant to anticancer therapies that do not directly target the PD-1/PD-L1 axis, such as, for example, immune-check point inhibitors targeting CTLA4.

Our results suggest the main mechanism responsible for aberrant glycosylation and decreased expression of PD-L1 with metformin and 2DG in TNBC cells is AMPK independent direct inhibition of N-glycosylation by 2DG with subsequent ER degradation, as neither A 769662 nor rapamycin significantly lowered glycosylated PD-L1 levels. Additionally, PD-L1 levels in Met + 0.6DG treated cells were normalized by mannose while the AMPK inhibitor compound C had no such effect (Figure 7F). We speculate that metformin treatment potentiates the effect of 2DG on protein N-glycosylation by depleting the pools of glycolysis intermediates, as observed in our previous work [54].

As T cells are the primary targets for immune suppression through the PD-L1/PD-1 axis, we next evaluated the effects of metformin and 2DG on PD-1 expression in activated Jurkat cells. In parallel, we also measured their PD-L1 expression as Jurkat cells are themselves of malignant origin. Metformin and 2DG both reduced PD-1 as well as PD-L1 expression on activated Jurkat cells in a dose-dependent manner. We also obtained reduced expression of CD69 for metformin, 2DG and their combinations, indicating blocked Jurkat cell activation with a similar pattern as for PD-1 and PD-L1. Additionally, Jurkat cells did not express PD-1 or PD-L1 without activation. The observed reduction in PD-1 and PD-L1 expression was therefore partly a result of blocked activation. However, the effect of 2DG on PD-1 compared to CD69 expression was stronger. This is evident from the ratio of relative PD-1 and CD69 fluorescence which was significantly decreased for 4.8 mM 2DG treatment, with 0.6 mM 2DG showing a similar trend (Figure 9D). Additionally, both PD-1 and PD-L1 expression were significantly reduced by tunicamycin, despite no effect on CD69 expression at 50 ng/mL. This is in agreement with other studies showing the importance of glycosylation for PD-1 stability [79]. Conversely, AMPK activator A 769662 also reduced PD-1 and PD-L1 expression despite only slightly reduced CD69 expression. Furthermore, the effector functions of activated Jurkat cells (as measured by IL-2 and IFN-γ secretion) were at least partially preserved for 4.8DG, Met and even Met + 0.6DG treatments (Figure 9E,F). Low (0.6 mM) 2DG and tunicamycin showed no effect on IL-2; 0.6 mM 2DG even vastly increased IFN-γ secretion, which potentially can be explained by the effect of ER stress on Nf-κB activation [80]. Taken together, these results imply that while blocked activation is likely the main mechanism of reduced PD-1 and PD-L1 expression, AMPK activation and inhibition of glycosylation also likely play a significant role, particularly with 0.6 mM 2DG (Figure 10). These results also suggest that the inhibition of glycosylation could be a promising metabolic strategy to reduce T cell inhibitory signaling through PD-L1/PD-1 axis in the context of anti-tumor immune response without excessively suppressing T-cell activation and effector functions, but further work will be needed to confirm this.

## 4. Materials and Methods

### 4.1. Cell Culture and Treatments

MDA-MB-231, BT-549 and Jurkat cells were acquired from ATCC. MDA-MB-231 and BT-549 cells were maintained in ATCC-modified RPMI 1640 medium (Genaxxon Bioscience) supplemented with 10% FBS, 2 mM glutamine and 25 mM glucose. For BT-549 cells only, the medium was also supplemented with 1 mM pyruvate (Pyr) and 0.023 IU/mL insulin. For all experiments, MDA-MB-231 and BT-549 cells were seeded in complete RPMI 1640 with 25 mM glucose for 24 h, washed with isotonic NaCl, and subsequently grown in RPMI 1640 medium supplemented with 10% FBS, 2 mM glutamine and 5.6 mM or 0 mM glucose. For BT-549 cells only, both the seeding and experimental media were also supplemented with 1 mM Pyr and 0.023 IU/mL insulin. Jurkat cells were maintained in ATCC-modified RPMI 1640 medium with 25 mM glucose, 2 mM glutamine and 1 mM Pyr (Gibco, Thermo Fisher, Waltham, MA, USA), supplemented with 10% FBS. All experiments were performed in in RPMI 1640 medium (Genaxxon bioscience GmbH, Ulm, Germany) supplemented with 10% FBS, 2 mM glutamine and 5.6 mM or 0 mM glucose.

### 4.2. Flow Cytometry

Cells were seeded on 12-well culture plates and treated with 0.6 mM or 4.8 mM 2DG, 5 mM metformin or their combination for 24 h to 72 h with daily medium change. Rapamycin (1.0 μM), A 769662 (70 μM or 150 μM) and tunicamycin (50 ng/mL or 500 ng/mL) treatment were used as additional controls.

For determination of mitochondrial mass, cells were stained after 72 h treatment with 7.5 μM nonyl-acridine orange (NAO) for 15 min at 37 °C. For additional confirmation, some samples were also stained with 200 nM Mitotracker Orange^®^ for 30 min at 37 °C. Cells were then harvested, resuspended in PBS and their fluorescence measured on Attune NxT flow cytometer. For determination of dead cells, the cells were harvested and resuspended in PBS. Propidium iodide was added directly prior to the measurement on Attune NxT flow cytometer. For surface marker expression, cells were harvested by centrifugation (Jurkat) or replacing the culture medium with PBS + 2 mM EDTA for 5 min at room temperature and washing them with a pipette (MDA-MB-231 and BT-549). Cells were counted and 75,000 cells were stained with APC-conjugated anti-PD-L1 antibody (329707, Biolegend, San Diego, CA, USA), APC-conjugated anti-PD-1 (329908, Biolegend, San Diego, CA, USA) and Pacific Blue conjugated anti-CD69 (310920, Biolegend, San Diego, CA, USA) at room temperature for 20 min. Cells were then washed and analyzed on Attune NxT flow cytometer.

### 4.3. Determination of Total Cell Number

The total cell number was determined as already described [30] using a Countess cell counter (Invitrogen, Waltham, MA, USA). Briefly, floating and attached cells were separately collected, centrifuged and resuspended in a known volume of the medium. Cells were stained by Trypan blue and floating and attached cells were counted separately using Countess cell counter. The total number of cells was calculated as a sum of attached and floating cells. It was normalized to the number of seeded cells. In some experiments, the number of cells was determined spectrofluorimetrically by staining the DNA with Hoechst 33342 as previously described [45].

### 4.4. Transmission Electron Microscopy

MDA-MB-231 cells were seeded on 40 mm Petri dishes at 180,000 cells per dish and treated with 5 mM metformin for 48 h or 72 h. For cells treated with 4.8 mM 2DG or 5 mM metformin plus 0.6 mM 2DG in complete RPMI medium supplemented with 5.6 mM glucose for 72 h or 5 mM metformin for 48 h in complete medium supplemented with 0 mM glucose, the seeding density was increased to 240,000 cells per dish. For all samples, the medium was changed daily. Following treatment, the cells were fixed and prepared for transmission electron microscopy samples as described in [81]. Briefly, cells were fixed in a mixture of 2% (*v*/*v*) glutaraldehyde and 4% (*w*/*v*) paraformaldehyde in 0.1 M cacodylate buffer, pH 7.4, for 3 h at 4 °C. Cells were post-fixed in 1% osmium tetroxide in 0.1 M cacodylate buffer for 1 h and after dehydration embedded in resin.

Representative transmission electron micrographs of ultrathin sections were captured with TEM CM 100 (Philips Electron Optics, Eindhoven, The Netherlands) so that the part of the cell containing part of the nucleus and cytoplasm with mitochondria was visible. The mitochondria were counted and normalized to the measured area of cytoplasm on the micrographs. The length of individual mitochondria was determined as the distance between the furthest points of their outer membrane, and the average length of mitochondria was determined for each micrograph.

### 4.5. RNA Quantification by Quantitative Real-Time PCR

MDA-MB-231 cells were seeded on 6-well culture plates in complete RPMI medium with 25 mM glucose. After 24 h, the cells were washed with isotonic NaCl solution and the medium was changed to complete RPMI supplemented with 5.6 mM or 0 mM glucose. Cells were treated with 0.6 mM or 4.8 mM 2DG, 5 mM metformin or their combination for 24 h or 48 h. After treatment, attached and detached cell populations were harvested and washed twice in ice-cold PBS and snap frozen in liquid nitrogen. RNA was extracted from cells using TRI-reagent^®^ (Merck, Darmstadt, Germany) according to the manufacturer’s instructions. The purity and concentration of nucleic acids was determined using a Synergy™ 2 spectrophotometer (Biotek, Winooski, VT, USA). Integrity of RNA was checked using agarose gel electrophoresis and a 2100 Bioanalyzer Instrument (Agilent, Santa Clara, CA, USA) with a RNA 6000 Nanochip. RNA with RIN > 8 was transcribed into cDNA using a high-capacity cDNA reverse transcription kit (Thermo Fisher, Waltham, MA, USA). RNA nucleotide sequences of target genes *PPARGC1A*, *TFAM*, *HSPA5*, *XBP1S*, *XBP1*,* XBP1U* and the reference gene *B2M* were retrieved from the NCBI Nucleotide database (www.ncbi.nlm.nih.gov/nuccore/, accessed on 19 August 2019 for PPARGC1A, 16 July 2019 for *TFAM*, 11 June 2019 for *HSPA5*, 24 July 2019 for *XBP1*,* XBP1S*,* XBP1U* and 21 March 2011 for *B2M*). Isoform non-specific primers were hand-picked and designed using the Primer3 (https://bioinfo.ut.ee/primer3/) and IDT OligoAnalyzer™ Tool (eu.idtdna.com/calc/analyzer, accessed on 19 August 2019 for PPARGC1A, 16 July 2019 for *TFAM*, 11 June 2019 for *HSPA5*, 24 July 2019 for *XBP1*,* XBP1S*,* XBP1U* and 21 March 2011 for *B2M*). Primer sequences and gene ID are summarized in Table S1. All primers were synthesized by Sigma (Merck, Darmstadt, Germany). The reverse transcription quantitative polymerase chain reaction (qRT-PCR) gene expression assay was carried out using Lightcycler 480^®^ SYBR Green I Master Mix and QuantStudio 12K Flex Real-Time PCR System (Life Technologies, Waltham, MA, USA) according to the manufacturer’s instructions. Melting curves for each sample were analyzed after each run to confirm the specificity of amplification. Raw Ct values were obtained from three run-independent technical replicates for each sample. Normalization of raw data was performed using geometric averaging of reference gene and relative expression was calculated using −ΔΔCt method [82].

### 4.6. Western Blotting

MDA-MB-231 and Jurkat cells were seeded on 6-well plates at 240,000 cells (MDA-MB-231) or 500,000 cells/mL (Jurkat) per well and treated with 0.6 mM or 4.8 mM 2DG, 5 mM metformin or their combination for 24 h. Rapamycin (1.0 μM), A769662 (100 μM) and tunicamycin (50 ng/mL) treatment were used as additional controls. After treatment, cells were washed twice with ice-cold PBS and harvested in Laemmli buffer (62.5 mM Tris-HCl, pH 6.8, 2% (*w*/*v*) SDS, 10% (*v*/*v*) glycerol, 5% 2-mercaptoethanol, 0.002% bromophenol blue). Total protein concentration was determined by means of a Pierce 660 (Thermo Fisher) and samples, containing equal amount of proteins, were loaded on 11% or 15% polyacrylamide gel and separated using electrophoresis (Mini-protean tetra cell system, Bio Rad). Subsequently, proteins were transferred to PVDF membrane. Ponceau S (0.1% (*w*/*v*) Ponceau S in 5% (*v*/*v*) acetic acid) was used to evaluate the efficiency of the protein transfer and sample loading. Membranes were blocked in 5% (*w*/*v*) skimmed milk in TBS-T (20 mM Tris, 150 mM NaCl, 0.02% (*v*/*v*) Tween-20, pH 7.5), which was followed by overnight incubation in primary antibodies against P-S6RP (sc-293144, Santa Cruz Biotechnology, TX, USA), GAPDH (#2118, Cell Signaling, Danvers, MA, USA), LC3B (#3868, Cell Signaling), β-actin (#3700, Cell Signaling) or PD-L1 (#13684, Cell Signaling, Danvers, MA, USA) at 4 °C. After washing, membranes were incubated with the appropriate secondary horseradish peroxidase-conjugated anti-mouse or anti-rabbit antibody. Enhanced chemiluminescence using Agfa X-ray film was used to detect immuno-reactive proteins. ImageJ was used for densitometric analysis.

### 4.7. Seahorse Real-Time ATP Production Rate Assay and Mito Stress Assay

MDA-MB-231 cells were plated on Seahorse XFe24 cell culture microplates at 20,000 cells per well in complete RPMI 1640 medium (2 mM glutamine, 0 mM pyruvate, 10% FBS) with 25 mM glucose. For the Mito Stress Assay, cell seeding density was increased to 30,000 per well for 4.8 mM 2DG and 5 mM metformin + 0.6 mM 2DG treated cells. After 24 h, cells were washed with isotonic NaCl solution and the medium was replaced with complete RPMI 1640 medium (2 mM glutamine, 0 mM pyruvate, 10% FBS) supplemented with 5.6 mM or 0 mM glucose as indicated, and treated with 5 mM metformin, 0.6 mM or 4.8 mM 2-deoxy-D-glucose (2DG), or their combination for 48 h, with a medium renewal after 24 h. A 769662 (70 μM) was used as an additional control for Mito Stress Assay. After 48 h of treatment, the medium was replaced with the appropriate RPMI 1640-based Seahorse XF Glycolytic Rate Assay medium (2 mM glutamine, 1 mM HEPES, 0 mM pyruvate, 5.6 mM or 0 mM glucose) equilibrated to pH 7.4, with the same concentrations of metformin and 2DG as the treatment media. The oxygen consumption rate (OCR) and extracellular acidification rate (ECAR) were then measured on the Seahorse XFe24 analyzer (Seahorse Bioscience, North Billerica, MA, USA) and the ATP production rate from glycolysis and oxidative phosphorylation determined according to Seahorse Real-Time ATP Rate Assay (1.5 μM oligomycin, 0.5 μM rotenon/antimycin A) protocol or Mito Stress Assay (1.5 μM oligomycin, 1 μM FCCP, 0.5 μM rotenon/antimycin A) protocol. Results were normalized to the total DNA content as determined by Hoechst staining.

Jurkat cells were seeded on 24-well cell culture plates at 250,000 cells/mL and treated for 48 h with 5 mM metformin, 0.6 mM or 4.8 mM 2-deoxy-D-glucose (2DG) as indicated. A 769662 (70 μM) was used as an additional control. After treatment, cells were spun down and resuspended in Seahorse XF RPMI 1640-based Seahorse XF Glycolytic Rate Assay medium (2 mM glutamine, 1 mM HEPES, 0 mM pyruvate, 5.6 mM or 0 mM glucose) equilibrated to pH 7.4 and plated on Seahorse cell culture microplates covered with CellTak^®^ at 150,000 cells in 0.1 mL per well. Plates were spun down at 200 g for 1 min and incubated at 37 °C without CO_2_ for 15 min, after which 0.4 mL of the medium was added and after additional 30 min of incubation at 37 °C without CO_2_. The Seahorse Mito Stress Assay was then performed according to the manufacturer’s instructions using 1.5 μM oligomycin, 1.5 μM FCCP and 0.5 μM rotenone/antimycin A.

### 4.8. ELISA

Jurkat cells were activated with 25 ng/mL PMA and 1.0 μM ionomycin for 24 h, during which they were treated with metformin and/or 2DG as indicated. After treatment, supernatant was collected and the concentration of secreted IL-2 determined with human IL-2 ELISA kit (88-7025, Invitrogen, Waltham, MA, USA) and IFN-γ ELISA kit (88-7316, Invitrogen, Waltham, MA, USA) according to the manufacturer’s instructions.

### 4.9. Mitochondrial DNA Quantification

The mitochondrial DNA (mtDNA) content was quantified by qRT-PCR as described previously [83]. Briefly, DNA was extracted from cells using QIAamp DNA Mini Kit (Qiagen, Hilden, Germany) according to the manufacturer’s instructions. The quantity and quality of DNA were measured using Synergy™ 2 spectrophotometer (Biotek, Winooski, VT, USA). The qRT-PCR was carried out as for RNA quantification. The mitochondrial encoded nicotinamide adenine dinucleotide dehydrogenase 1 (MT-ND1) and nuclear DNA encoded hemoglobin beta (HBB) primers were described previously [83]. The mtDNA was quantified using the equation: 2 × 2^−∆Ct^ [84].

### 4.10. Statistical Analysis

Results were displayed as mean ± SEM of three biological replicates unless indicated otherwise. Unless stated otherwise, one-way ANOVA with Dunnett’s post hoc test was used to test statistical significance of results. In some cases, two-way ANOVA was used to test for synergism between metformin and 2DG treatment.

## 5. Conclusions

The multiple anticancer mechanisms of metformin and 2DG are still under investigation. In the present study, we found that the combined treatment with metformin and in vivo achievable concentration (0.6 mM) of 2-deoxyglucose (2DG) increased mitochondrial mass in MDA-MB-231 and BT-549 triple-negative breast cancer (TNBC). This was accompanied by an increase in the size rather than number of mitochondria, indicating a shift towards fused state of mitochondria. A similar but weaker effect on mitochondrial mass was observed for 2DG at higher concentration (4.8 mM), while glucose deprivation had in general only a small effect on mitochondria. The increase in mitochondrial mass resulted from increased mitochondrial biogenesis rather than mitophagy inhibition. Interestingly, AMPK activation or mTOR suppression alone were insufficient to trigger the mitochondrial biogenesis. The results suggest that strong energy stress together with the inhibition of protein N-glycosylation and resulting endoplasmic reticulum (ER) stress induced by 2DG and potentiated by metformin lead to an increase in mitochondria, probably as an adaptive response of cells to optimize their mitochondrial respiration.

Importantly, as both metformin and 2DG were suggested to have an immunomodulatory effect through the PD-1/PD-L1 axis, we further explored these effects. We found that the combined treatment with metformin and 2DG had a favorable effect on potential anti-tumor immunity by causing deglycosylation and reduced expression of PD-L1, a key immune checkpoint protein, in MDA-MB-231 cells. The effect was already present with 2DG alone and was potentiated by metformin co-treatment. In contrast with a previous report, we found that the main mechanism responsible was the inhibition of protein N-glycosylation rather than direct phosphorylation by AMPK [42]. The effect of 2DG was consistent across the cell lines, while the combined treatment with metformin and 0.6 mM 2DG actually increased PD-L1 levels in BT-549 cells. Interestingly, we found increased PD-L1 levels in low glucose conditions for both TNBC cell lines. This is important as glucose levels in the tumor environment are generally very low. Metformin and 2DG treatments also reduced PD-1 expression on activated Jurkat cells to a larger extent than they blocked activation or effector functions. Overall, the reduction in PD-L1 and PD-1 expression combined with partially preserved Jurkat effector functions could be beneficial in the context of cancer immunotherapy that does not directly target the PD-1/PD-L1 axis.

Altogether, the combination of metformin and 2DG exhibits major effects on cancer cells beyond reduced proliferation and energy stress mainly through the effect on N-glycosylation, which could be explored for combinational therapies in the future.

## Data Availability

The data presented in this study are available in the article or in the Supplementary Material.

## Supplementary Materials

The following are available online at https://www.mdpi.com/article/10.3390/cancers14051343/s1, Figure S1: The effect of metformin and 2DG treatment on mitochondrial mass and cell death TNBC cells, Figure S2: The effect of metformin and 2DG treatment on MDA-MB-231 cell morphology and survival., Figure S3: The effect of metformin and 2DG on mitochondrial DNA copy number, Figure S4: The effect of metformin and 2DG on maximal respiratory capacity and glycolysis in MDA-MB-231 cells, Figure S5: The effect of metformin and 2DG on the mTOR pathway, Figure S6: Relative mRNA expression of mitochondrial biogenesis regulators in MDA-MB-231 cells, Figure S7: Relative mRNA expression of markers of ER stress and AMPK activation in MDA-MB-231 cells, Figure S8: The effect of metformin and 2DG on PD-L1 expression in TNBC cells, Figure S9: The effect of metformin treatment on Jurkat cell mitochondria and survival, Figure S10: The effect of metformin and 2DG on Jurkat cell activation, PD-L1 expression and effector functions, Figure S11: Original western blot images, Table S1: Primer sequences and gene ID of target and reference genes.
