# Supplementary material for: Dual Effect of Combined Metformin and 2-Deoxy-D-Glucose Treatment on Mitochondrial Biogenesis and PD-L1 Expression in Triple-Negative Breast Cancer Cells
Source: Cancers (Basel). 2022 Mar 5;14(5):1343. doi: 10.3390/cancers14051343 (PMC8909901; doi:10.3390/cancers14051343)
Supplement: Supplementary file 1 [file cancers-14-01343-s001.zip › cancers-1593975-SI.pdf]

cancers

MDPI

Article

Dual Effect of Combined Metformin and 2-Deoxy-D-Glucose Treatment on Mitochondrial Biogenesis and PD-L1 Expression in Triple-Negative Breast Cancer Cells

Jernej Repas, Mateja Zupin, Maja Vodlan, Peter Veranič, Boris Gole, Uroš Potočnik and Mojca Pavlin

Table S1. Primer sequences and gene ID of target and reference genes.

Gene Name	Gene ID	Forward Primer 5′–3′	Reverse Primer 5′–3′
PPARGC1A	10891	AAAAGCCACAAAGACGTCCC	TGTTGGTTTGGCTTGTAAGTGT
TFAM	7019	GCTCAGAACCCAGATGCAAA	TATATACCTGCCACTCCGCC
HSPA5	3309	GAAGAGCTCAACATGGATCTGT	CATCTGGGTTTATGCCACGG
XBP1	7494	AGCTCAGACTGCCAGAGATC	TCACTTCATTCCCCTTGGCT
XBP1S	7494+	CTGAGTCCGCAGCAGGTG	ACTGACAGAGAAAGGGAGGC
XBP1U	7494++	CGAATGAGTGAGCTGGAACA	GTAGTCTGAGTGCTGCGGA
B2M	567	TTCTGGCCTGGAGGCTATC	TCAGGAAATTTGACTTTCCATTC

+ transcript variant 2, ++ transcript variant 1.

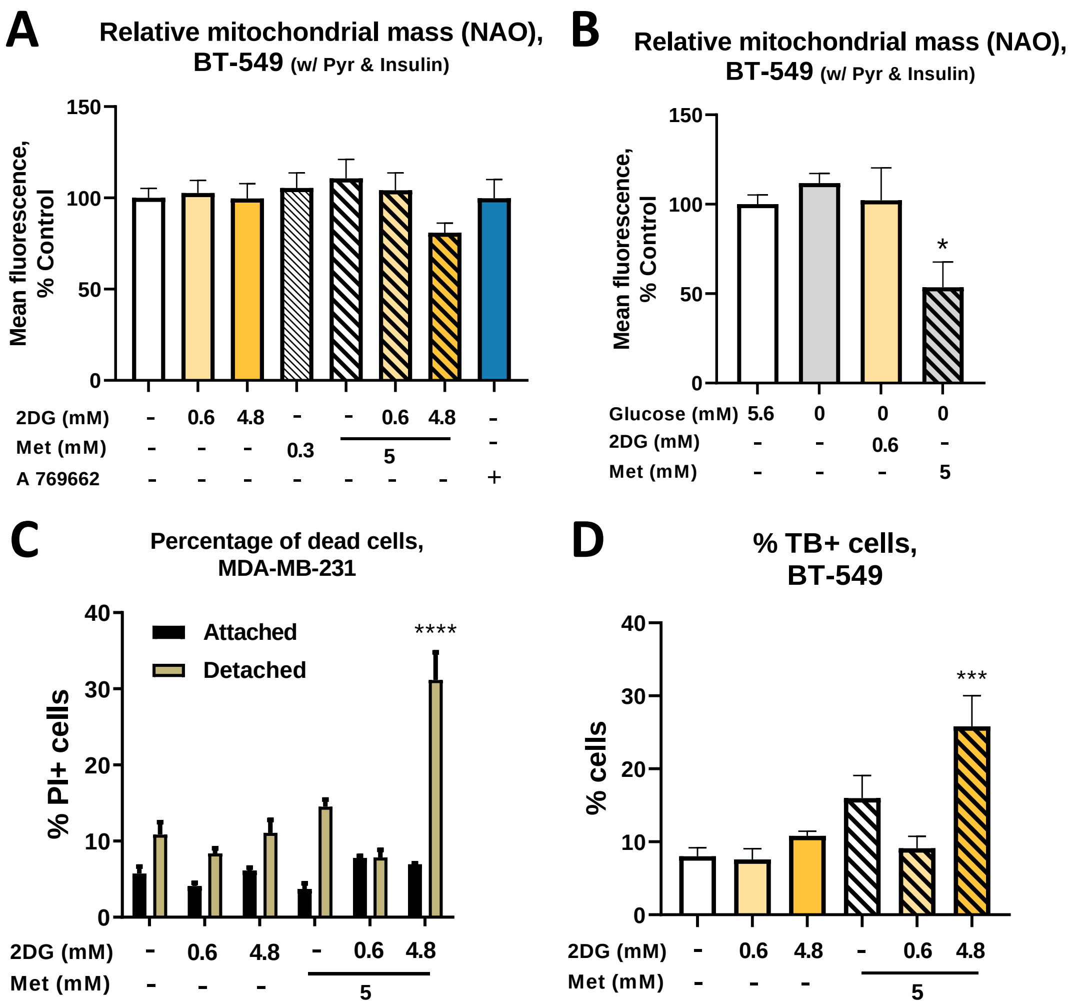

Figure S1. The effect of metformin and 2DG treatment on mitochondrial mass and cell death TNBC cells. (**A,B,D**) BT-549 cells were grown in medium supplemented with pyruvate and insulin and either 5.6 mM (**A, D**) or 0 mM glucose with daily medium change for 72 h. Mitochondrial mass was determined by NAO staining (**A–B**) and the percentage of dead cells by trypan blue staining and direct counting (**D**). (**C**) MDA-MB-231 cells were treated as indicated for 72 h in medium with 5.6 mM glucose. Attached and detached populations were separated, stained with propidium iodide (PI) and counted with flow cytometry. Mean ± SEM is shown for three independent experiments. *$p < 0.05$, ***$p < 0.001$,**** $p < 0.0001$ as determined by ANOVA.

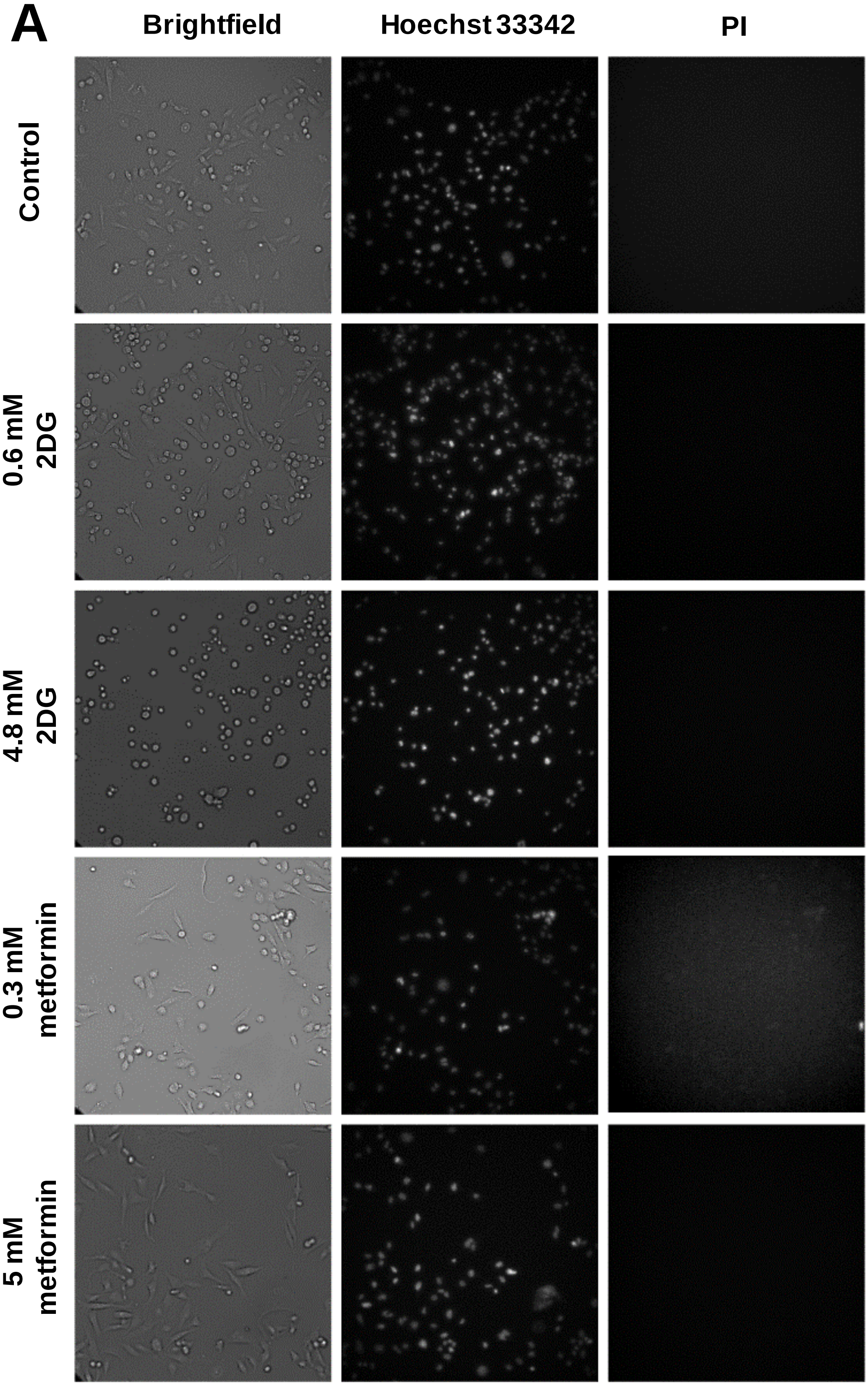

Figure S2. The effect of metformin and 2DG treatment on MDA-MB-231 cell morphology and survival. MDA-MB-231 cells were treated for 72 h in medium supplemented with 5.6 mM (**A**) or 0 mM (**B**) glucose as indicated, stained with Hoechst 33342 and PI and representative bright field micrographs captured.

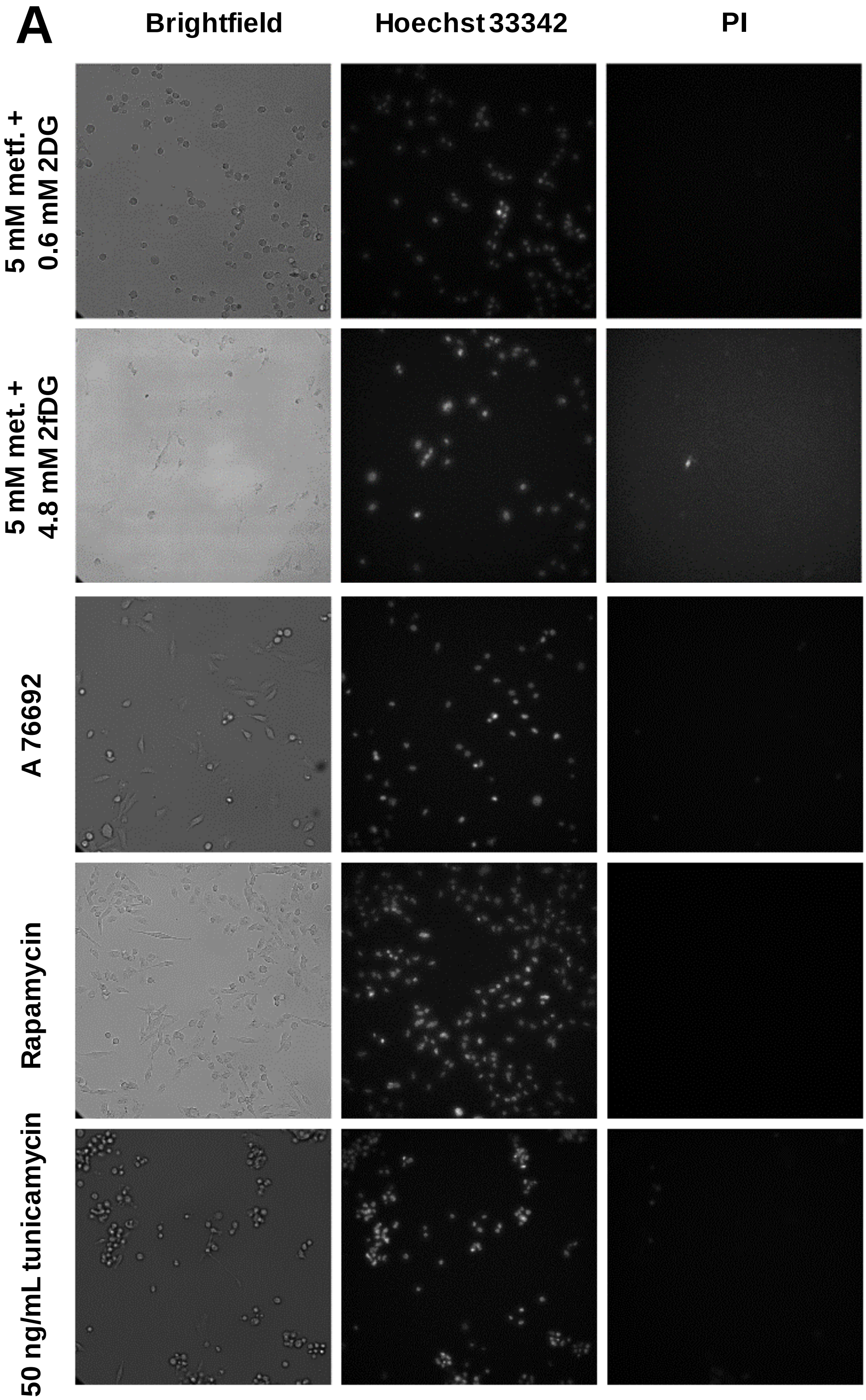

Figure S2. (continued). The effect of metformin and 2DG treatment on MDA-MB-231 cell morphology and survival. MDA-MB-231 cells were treated for 72 h in medium supplemented with 5.6 mM (**A**) or 0 mM (**B**) glucose as indicated, stained with Hoechst 33342 and PI and representative bright field micrographs captured.

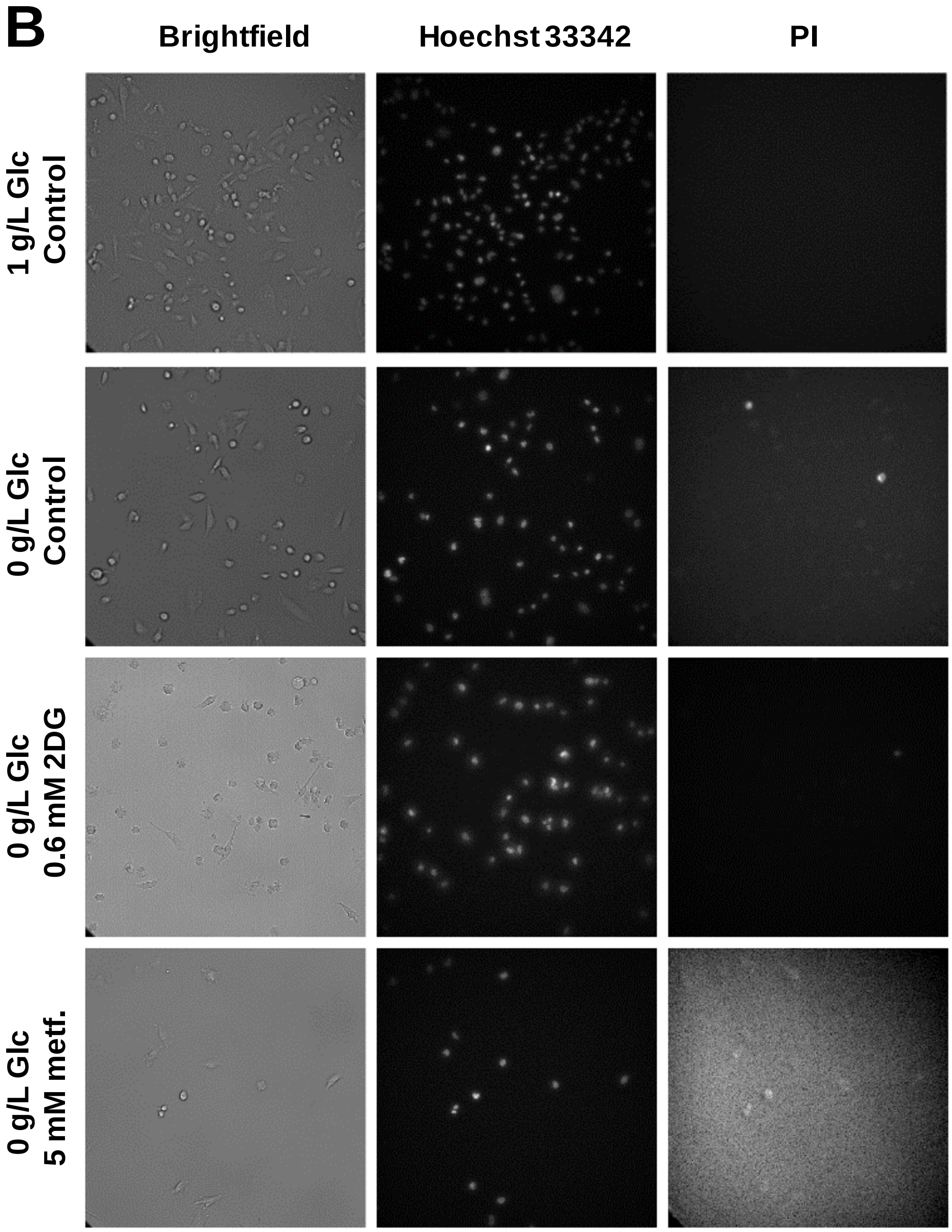

Figure S2. (continued). The effect of metformin and 2DG treatment on MDA-MB-231 cell morphology and survival. MDA-MB-231 cells were treated as indicated for 48 h in medium supplemented with 5.6 mM (**A**) or 0 mM (**B**) glucose with daily medium change, stained with Hoechst 33342 and PI and representative bright field micrographs captured.

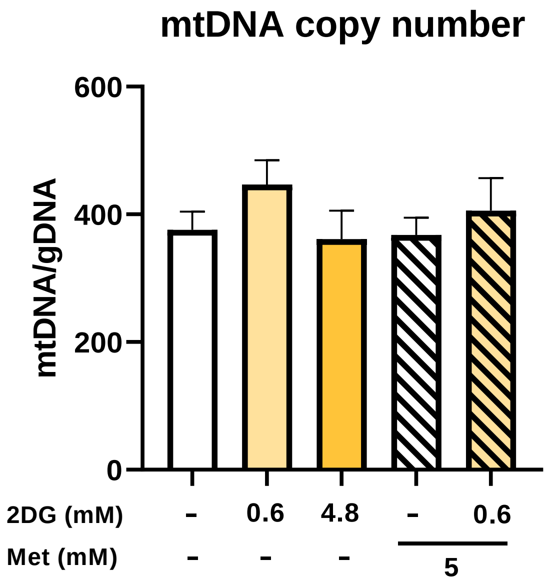

Figure S3. The effect of metformin and 2DG on mitochondrial DNA copy number. MDA-MB-231 cells were treated for 48 h with the indicated compounds in RPMI medium supplemented with 5.6 mM glucose as indicated, with daily medium change. Following treatment, the mitochondrial DNA content was determined and normalized to genome copy number. The mean ± SEM for four independent experiments is shown.

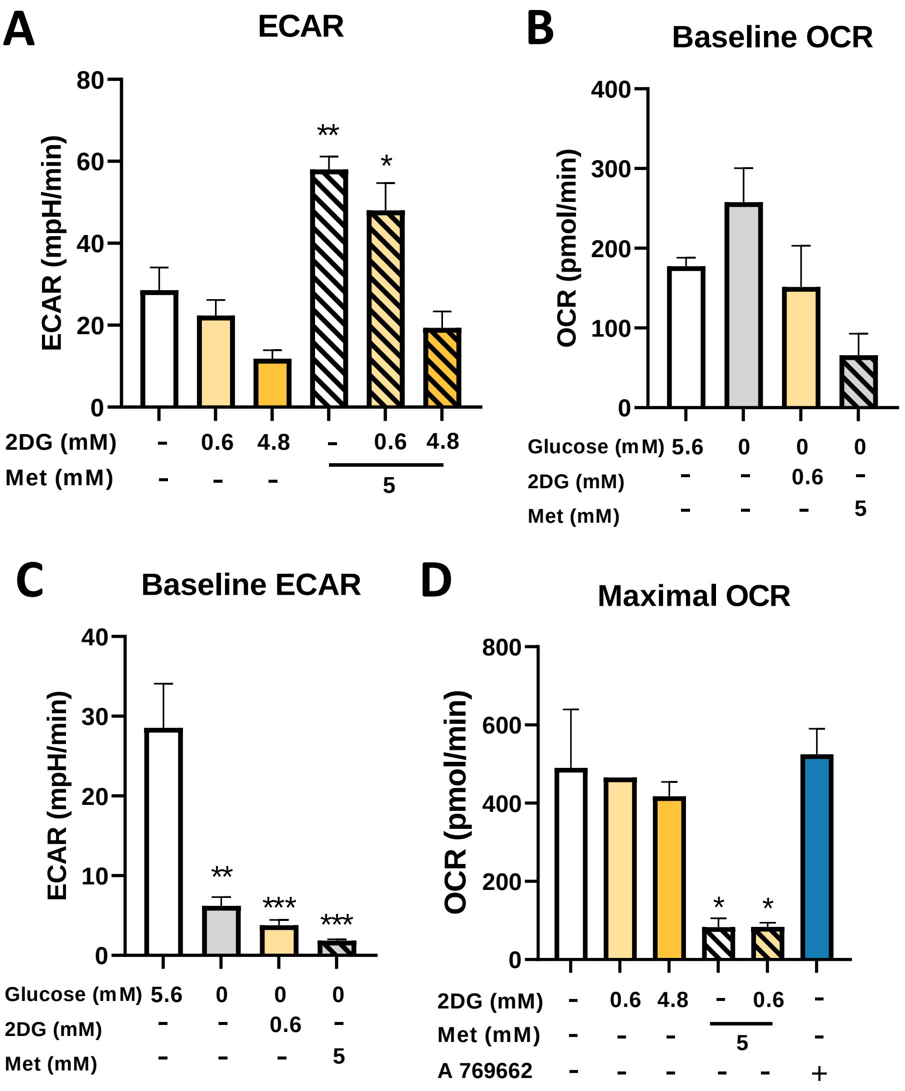

Figure S4. The effect of metformin and 2DG on maximal respiratory capacity and glycolysis in MDA-MB-231 cells. MDA-MB-231 cells were treated for 48 h with the indicated compounds in RPMI medium with 5.6 mM or 0 mM glucose as indicated, with daily medium change. Following treatment, the maximum oxygen consumption rate (OCR) following FCCP injection and baseline extracellular acidification rate (ECAR) were measured with Seahorse XFe24 analyzer using the Mito Stress Assay. The results were corrected for cell number and normalized to untreated control. The mean ± SEM two independent experiments is shown. *$p < 0.05$, **$p < 0.01$, ***$p < 0.001$ as determined by ANOVA.

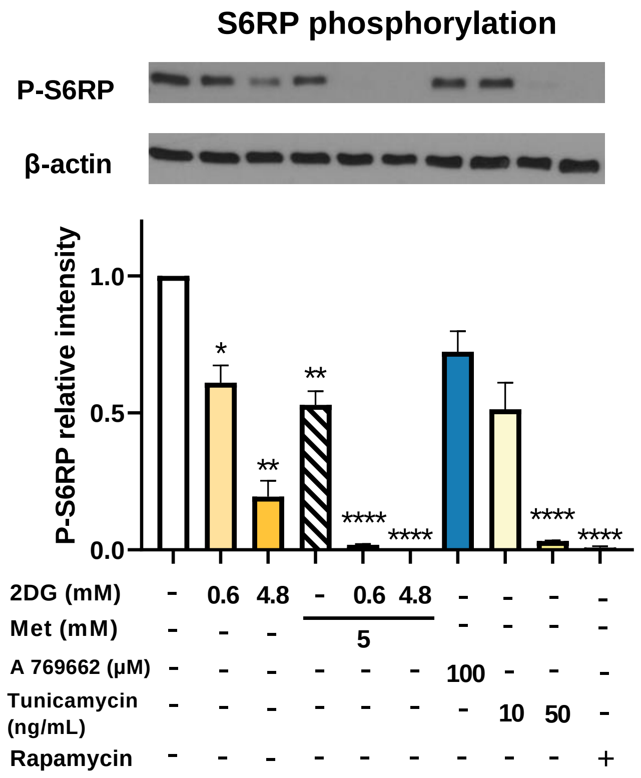

Figure S5. The effect of metformin and 2DG on mTOR pathway. MDA-MB-231 cells were treated with metformin and 2DG for 24 h after which cells were lysed and S6RP phosphorylation determined by western blot. * $p < 0.05$, ** $p < 0.01$, **** $p < 0.0001$ as determined by ANOVA.

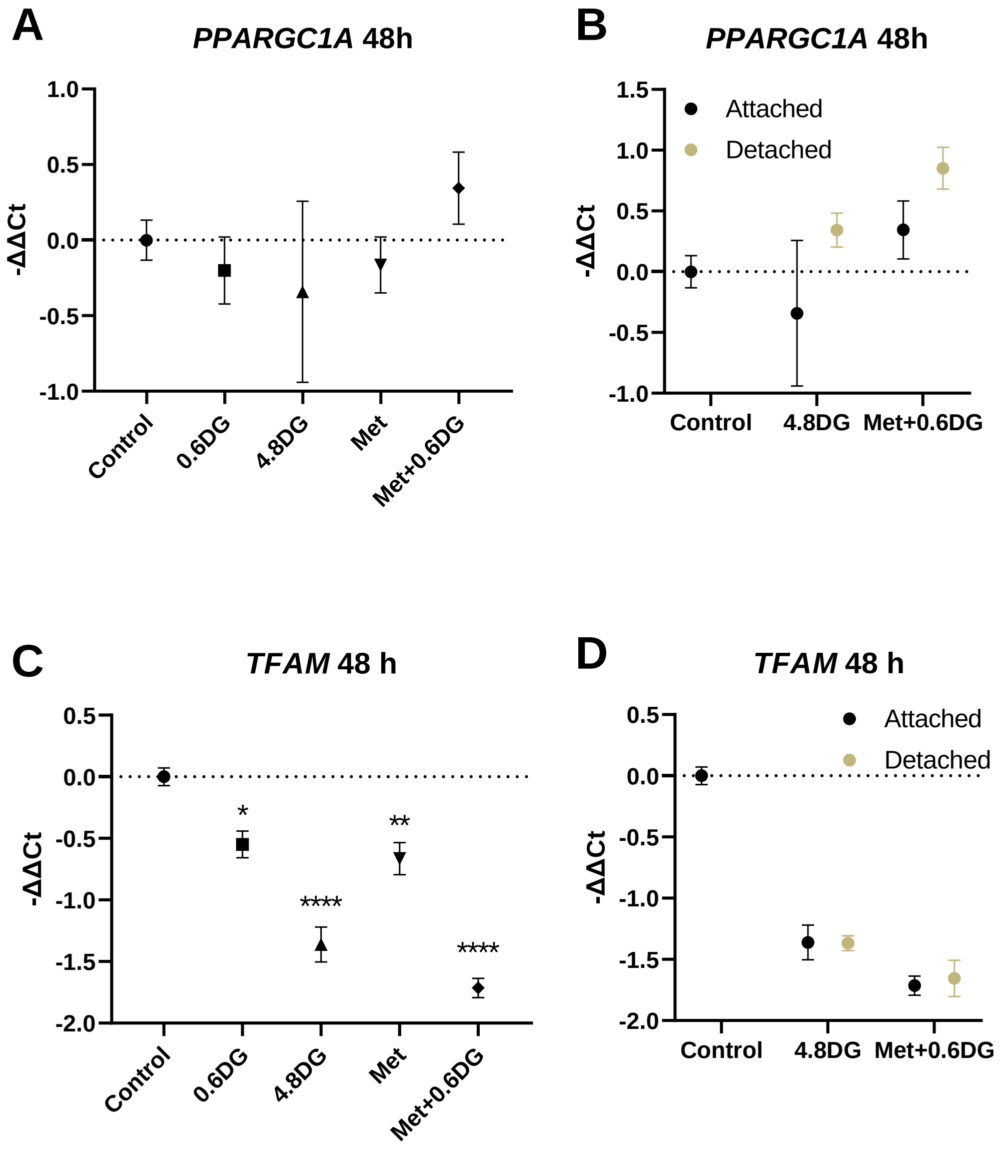

Figure S6. Relative mRNA expression of mitochondrial biogenesis regulators in MDA-MB-231 cells. MDA-MB-231 cells were treated for 48 h with the indicated compounds in RPMI medium supplemented with 5.6 mM glucose with daily medium change. Following treatment, attached (**A–D**) and detached (**B, D**) cells were collected separately and lysed, and relative mRNA abundance of transcripts for *PPARGC1A* (**A, B**) and *TFAM* (**C, D**) was determined with real-time PCR. Results were calculated as fold change compared to control cells. The mean ± SEM of 4 independent experiments is shown. *$p < 0.05$, **$p < 0.01$, ****$p < 0.0001$ as determined by ANOVA.

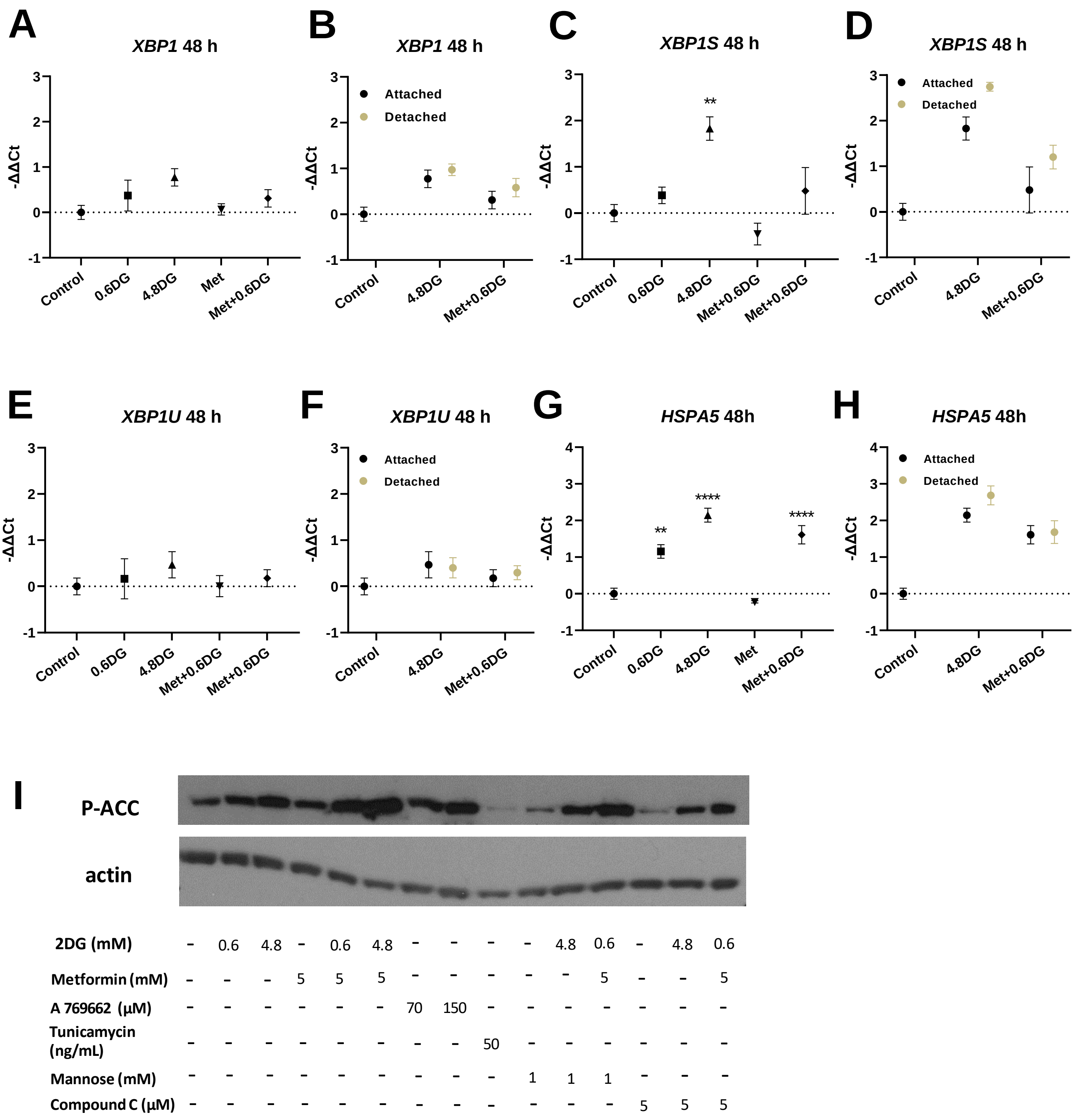

Figure S7. Relative mRNA expression of markers of ER stress and AMPK activation in MDA-MB-231 cells. MDA-MB-231 cells were treated for 48 h with the indicated compounds in RPMI medium supplemented with 5.6 mM glucose with daily medium change. (**A–H**) Following treatment, attached (**A–H**) and detached (B, D, F, H) cells were collected separately and lysed, and relative mRNA abundance of transcripts for total *XBP1* (**A, B**), spliced isoform *XBP1S* (**C, D**) and isoform *XBP1U* (**E, F**) as well as *HSPA5* (**G, H**) was determined with real-time PCR. Results were calculated as fold change compared to control cells. The mean ± SEM of 4 independent experiments is shown. (**I**) Western blot analysis of AMPK activations as measured by acetyl-CoA carboxylase phosphorylation. One representative blot from three independent experiments is shown. $^{**}p < 0.01$, $^{****}p < 0.0001$ as determined by ANOVA.

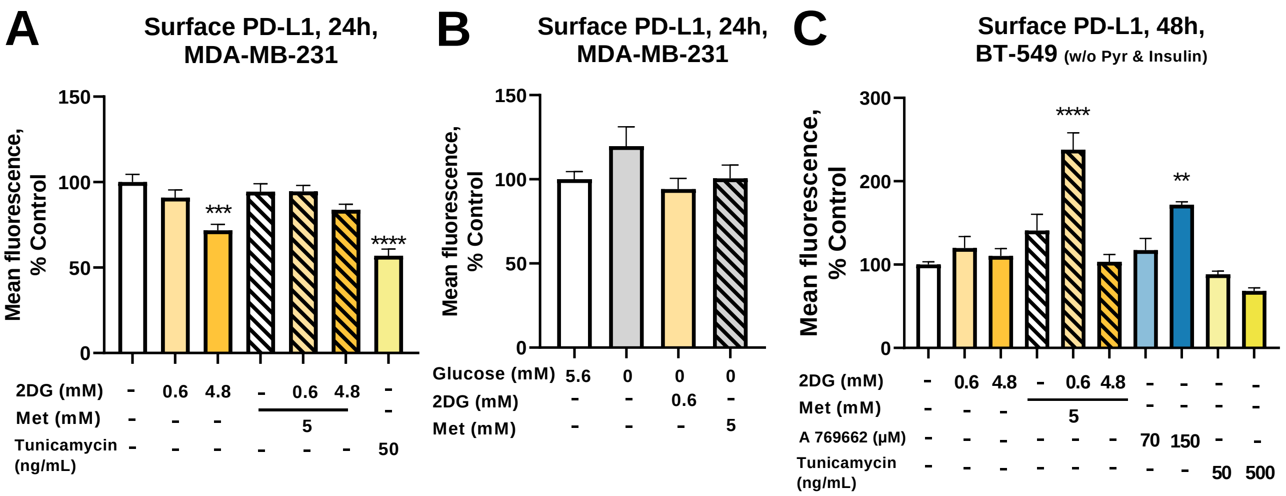

Figure S8. The effect of metformin and 2DG on PD-L1 expression in TNBC cells. (**A–B**) MDA-MB-231 cells were grown in the RPMI medium supplemented with 5.6 mM (**A**) or 0 mM (**B**) glucose and treated with indicated compounds for 24 h. (**C**) BT-549 cells were adapted to medium without pyruvate and insulin and treated with metformin and 2DG for 48 h with daily medium change. Surface PD-L1 expression was determined with flow cytometry. Mean ± SEM is shown for two (**C**) or three (**A–B**) independent experiments. ** $p < 0.01$, *** $p < 0.001$, **** $p < 0.0001$ as determined by ANOVA.

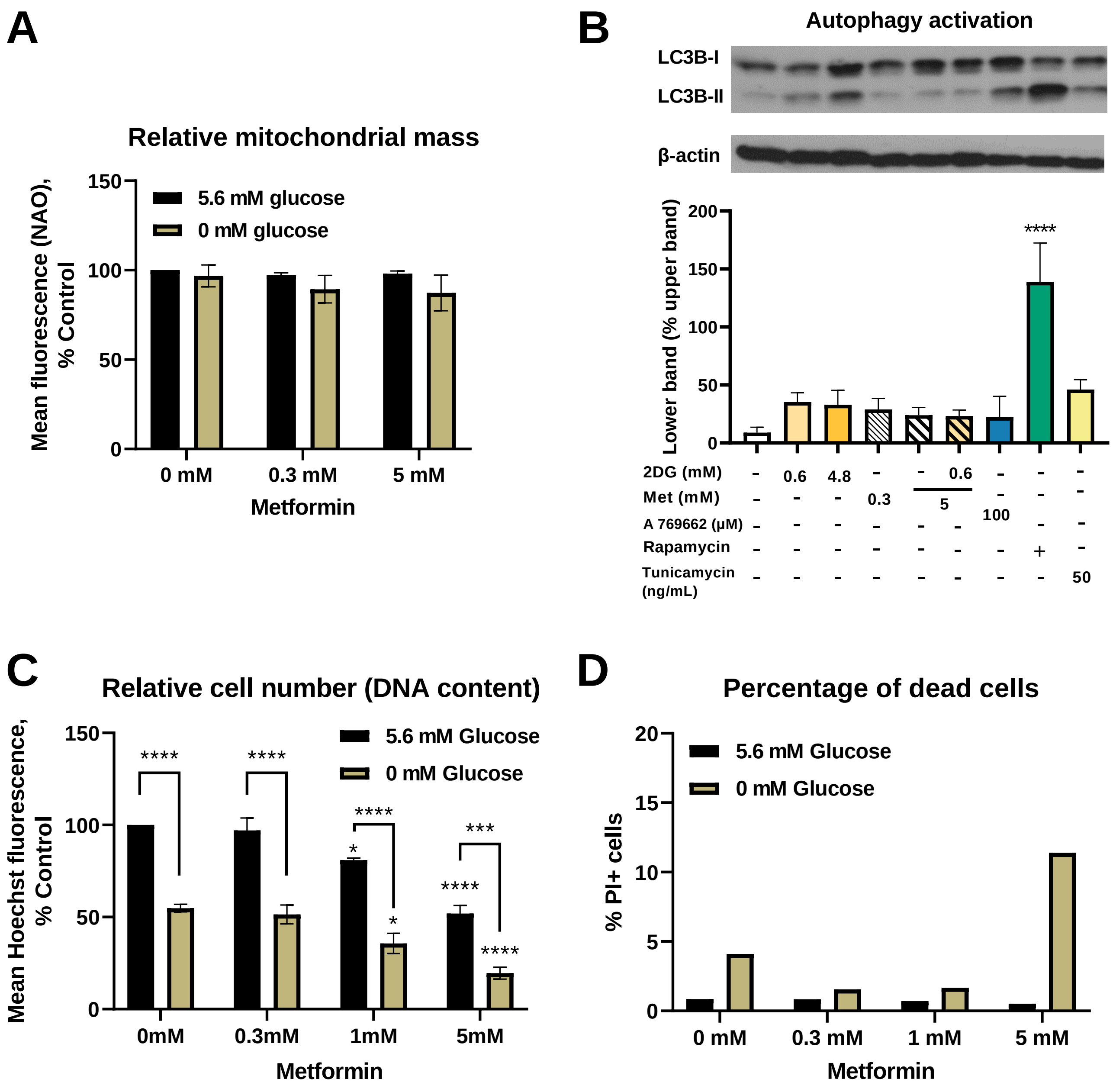

Figure S9. The effect of metformin treatment on Jurkat cell mitochondria and survival. (**A, C–D**) Jurkat cells were treated with metformin for 72 h in medium supplemented with 5.6 mM or 0 mM glucose as indicated. Relative mitochondrial mass was determined by NAO staining and flow cytometry (**A**). Cell number was determined by Hoechst 33342 staining and measuring the total DNA content determined (**C**). The percentage of dead (PI+) cells was determined with flow cytometry (**D**). (B) Jurkat cells were treated with metformin and 2DG for 24 h and the conversion of LC3B-I to LC3B-II determined by western blot. Mean ± SEM is shown for three independent experiments (**A–C**). *$p < 0.05$, ***$p < 0.001$, ****$p < 0.0001$ as determined by one way (**B**) or two-way (**A, C**) ANOVA.

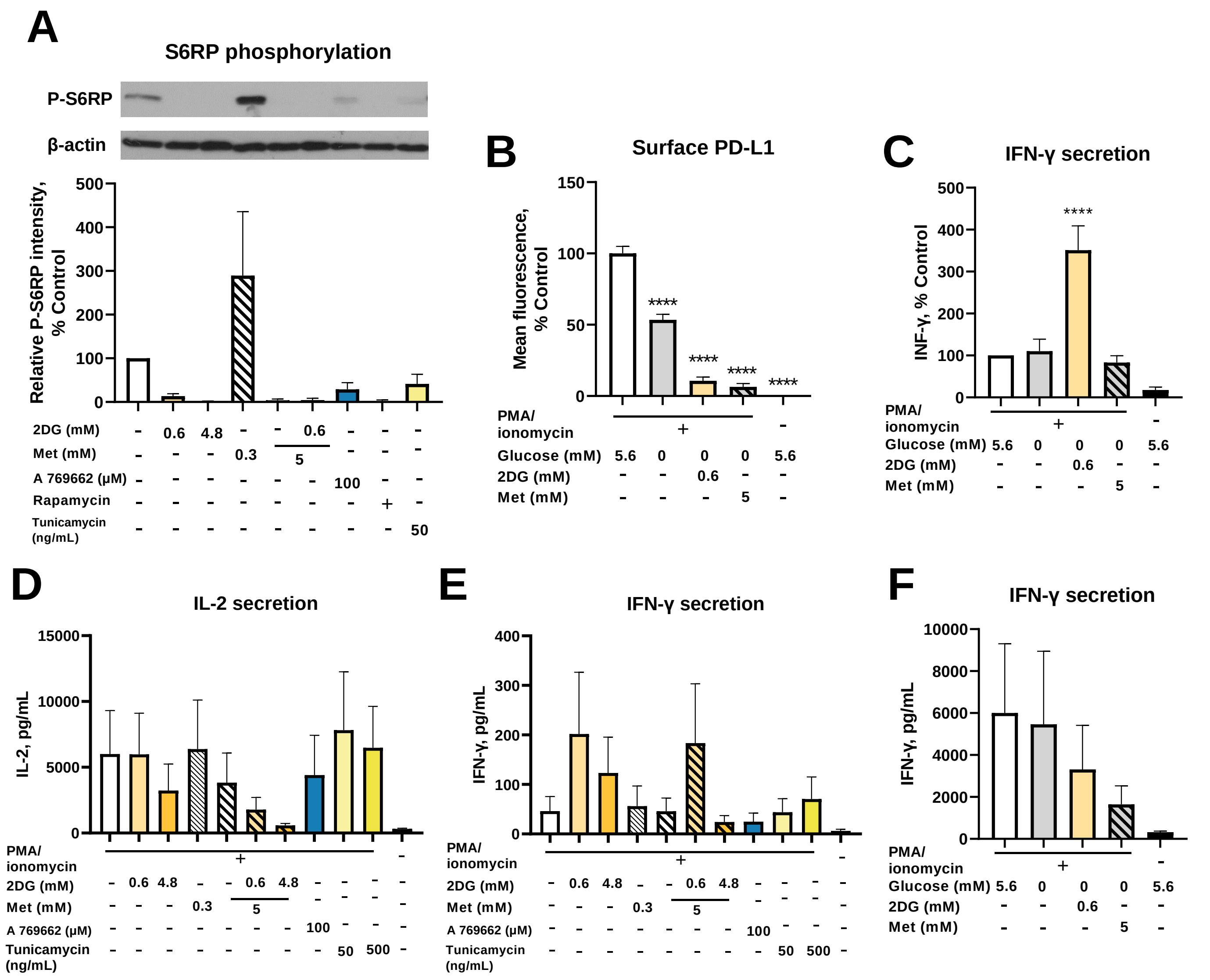

Figure S10. The effect of metformin and 2DG on Jurkat cell activation, PD-L1 expression and effector functions. (**A**) Jurkat cells were treated with metformin and 2DG as indicated for 24 h and the mTOR pathway activity was determined by measuring S6RP phosphorylation with western blot. (**B–F**) The cells were activated with 1.0 µM ionomycin and 25 ng/mL PMA in medium supplemented with 5.6 mM or 0 mM glucose and concurrently treated with metformin and 2DG. PD-L1 expression was determined by flow cytometry and IL-2 or IFN-γ secretion by ELISA. Mean ± SEM is shown for three (**A–C, E–F**) or four (**D**) independent experiments (**A–C**). ****$p < 0.0001$ as determined by ANOVA.

LC3B, MDA-MB-231 cells, Figure 5A

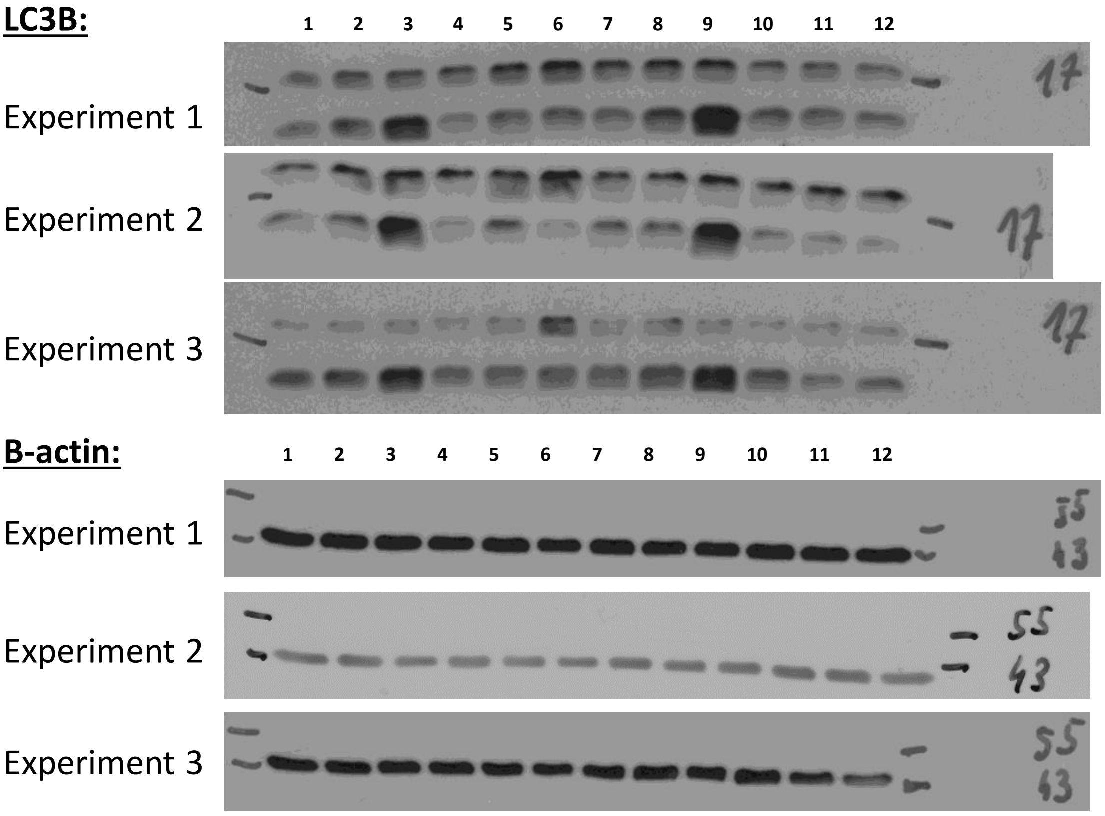

Lane	Sample
1	Control
2	0.6 mM 2DG
3	4.8 mM 2DG
4	5 mM Metformin
5	5 mM Metformin + 0.6 mM 2DG
6	5 mM Metformin + 4.8 mM 2DG
7	100 µM A 769662
8	10 ng/mL tunicamycin
9	50 ng/mL tunicamycin
10	1.0 µM rapamycin
11	0.1 µM rapamycin
12	5 µM rapamycin

Figure S11. Original western blot images.

P-S6RP, MDA-MB-231 cells, Figure S5

P-S6RP:

1 2 3 4 5 6 7 8 9 10 11 12

Experiment 1

Experiment 2

Experiment 3

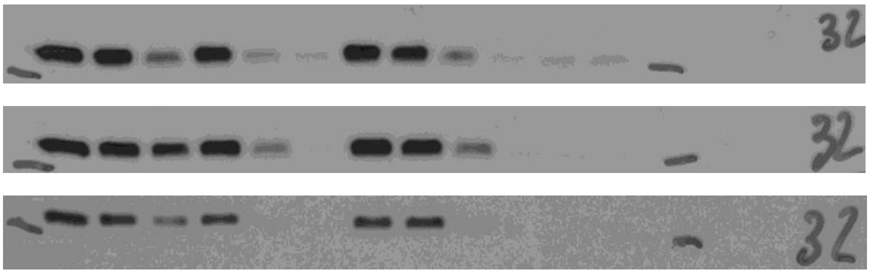

B-actin:

1 2 3 4 5 6 7 8 9 10 11 12

Experiment 1

Experiment 2

Experiment 3

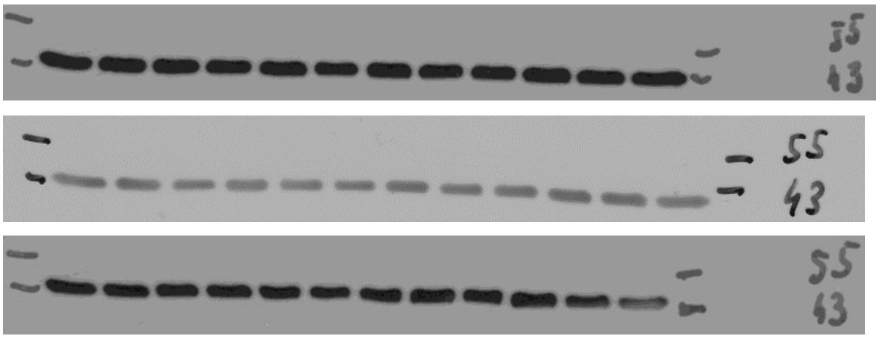

Lane	Sample
1	Control
2	0.6 mM 2DG
3	4.8 mM 2DG
4	5 mM Metformin
5	5 mM Metformin + 0.6 mM 2DG
6	5 mM Metformin + 4.8 mM 2DG
7	100 µM A 769662
8	10 ng/mL tunicamycin
9	50 ng/mL tunicamycin
10	1.0 µM rapamycin
11	0.1 µM rapamycin
12	5 µM rapamycin

Figure S11. Original western blot images (continued).

PD-L1 expression, MDA-MB-231 cells, Figure 7A (part 1)

PD-L1

Experiment 1

Experiment 2

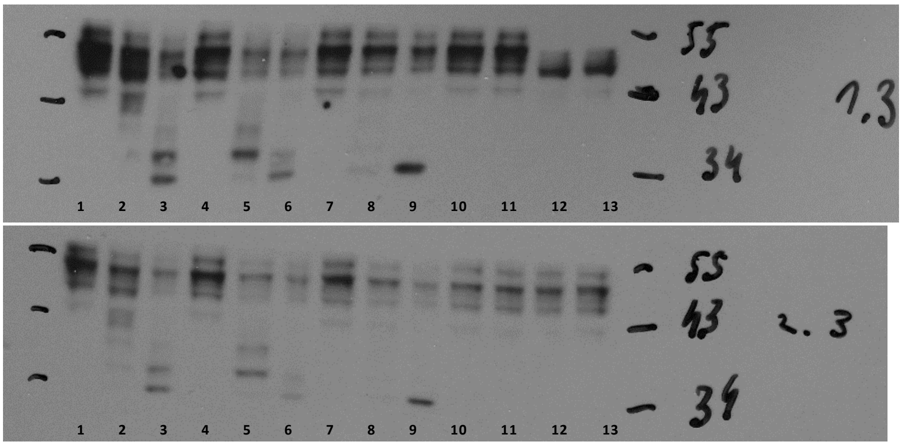

GAPDH

Experiment 1

Experiment 2

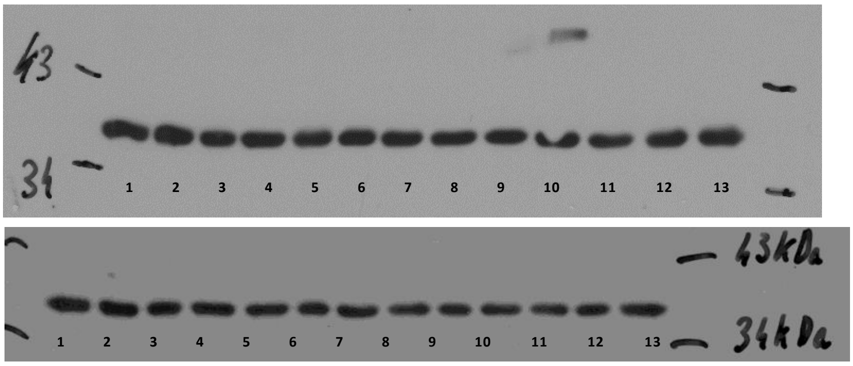

Experiment 1

Lane	Sample
1	Control
2	0.6 mM 2DG
3	4.8 mM 2DG
4	5 mM Metformin
5	5 mM Metformin + 0.6 mM 2DG
6	5 mM Metformin + 4.8 mM 2DG
7	100 µM A 769662
8	10 ng/mL tunicamycin
9	50 ng/mL tunicamycin
10	1.0 µM rapamycin
11	0.1 µM rapamycin
12	BT-549, Control
13	BT-549, 5 mM Metformin

Experiment 2

Lane	Sample
1	Control
2	0.6 mM 2DG
3	4.8 mM 2DG
4	5 mM Metformin
5	5 mM Metformin + 0.6 mM 2DG
6	5 mM Metformin + 4.8 mM 2DG
7	100 µM A 769662
8	10 ng/mL tunicamycin
9	50 ng/mL tunicamycin
10	1.0 µM rapamycin
11	0.1 µM rapamycin
12	5 µM rapamycin
13	Control

Figure S11. Original western blot images (continued).

PD-L1 expression, MDA-MB-231 cells, Figure 7A (part 2)

PD-L1: Experiment 3, Experiment 4

GAPDH: Experiment 3, Experiment 4

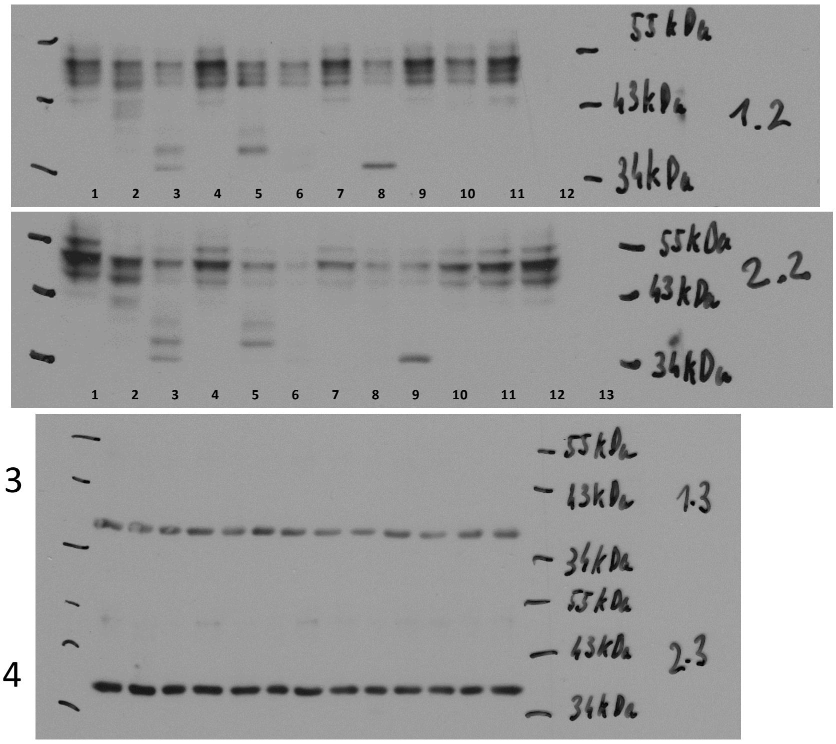

Experiment 3

Lane	Sample
1	Control
2	0.6 mM 2DG
3	4.8 mM 2DG
4	5 mM Metformin
5	5 mM Metformin + 0.6 mM 2DG
6	5 mM Metformin + 4.8 mM 2DG
7	100 µM A 769662
8	50 ng/mL tunicamycin
9	1.0 µM rapamycin
10	0 g/L Glc, Control
11	0 g/L Glc, 5 mM Metformin
12	300 µM A 769662
13	Laemmli buffer

Experiment 4

Lane	Sample
1	Control
2	0.6 mM 2DG
3	4.8 mM 2DG
4	5 mM Metformin
5	5 mM Metformin + 0.6 mM 2DG
6	5 mM Metformin + 4.8 mM 2DG
7	100 µM A 769662
8	10 ng/mL tunicamycin
9	50 ng/mL tunicamycin
10	1.0 µM rapamycin
11	5 µM rapamycin
12	0.1 µM rapamycin
13	Laemmli buffer

Figure S11. Original western blot images (continued).

PD-L1 expression, MDA-MB-231 cells, Figure 7D

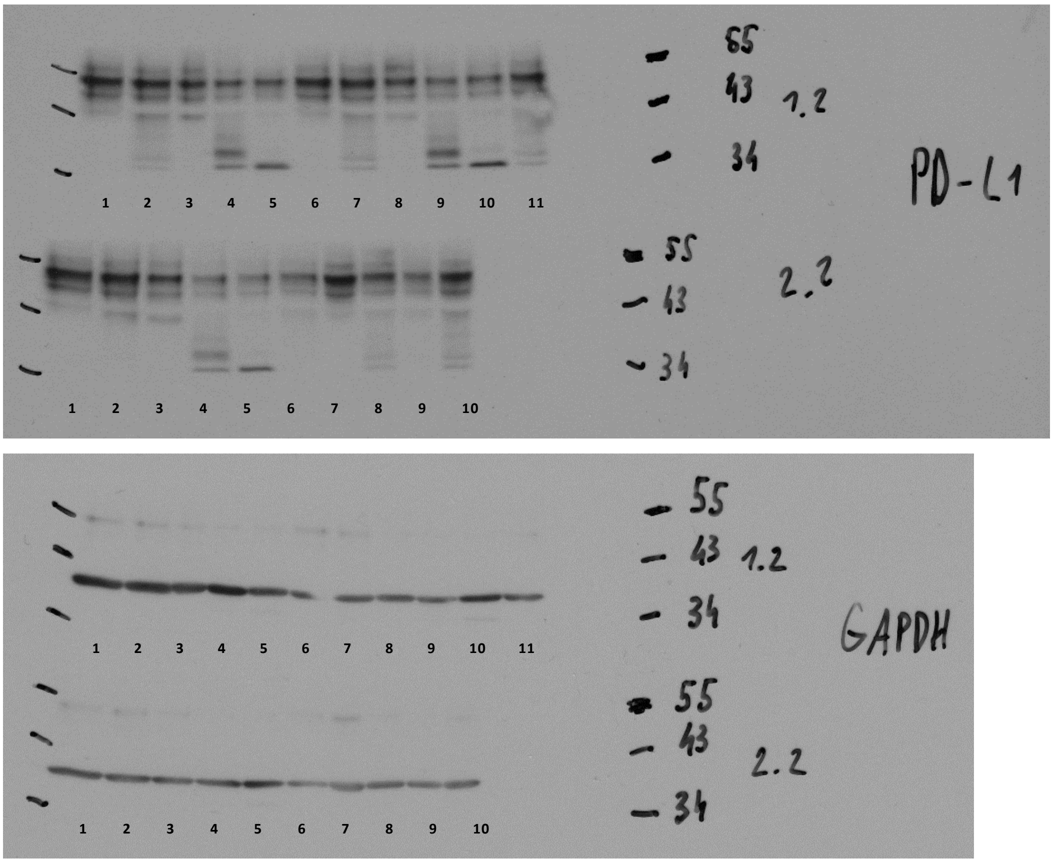

Gel 1 (top)

Lane	Sample	Independent experiment
1	1 g/L Glc, Control	
2	0 g/L Glc, Control	
3	0 g/L Glc, 5 mM metformin	1
4	0 g/L Glc, 0.6 mM 2DG	
5	50 ng/mL tunicamycin	
6	1 g/L Glc, Control	
7	0 g/L Glc, Control	
8	0 g/L Glc, 5 mM metformin	2
9	0 g/L Glc, 0.6 mM 2DG	
10	50 ng/mL tunicamycin	
11	0 g/L Glc, 0.3 mM metformin	

Gel 2 (bottom)

Lane	Sample	Independent experiment
1	1 g/L Glc, Control	
2	0 g/L Glc, Control	3
3	0 g/L Glc, 5 mM metformin	
4	0 g/L Glc, 0.6 mM 2DG	
5	50 ng/mL tunicamycin	2
6	0 g/L Glc, 0.3 mM metformin	3
7	1 g/L Glc, Control	
8	0 g/L Glc, Control	pilot
9	0 g/L Glc, 5 mM metformin	
10	0 g/L Glc, Control	

Figure S11. Original western blot images (continued).

ACC phosphorylation, MDA-MB-231 cells, Figure S7I (part 1)

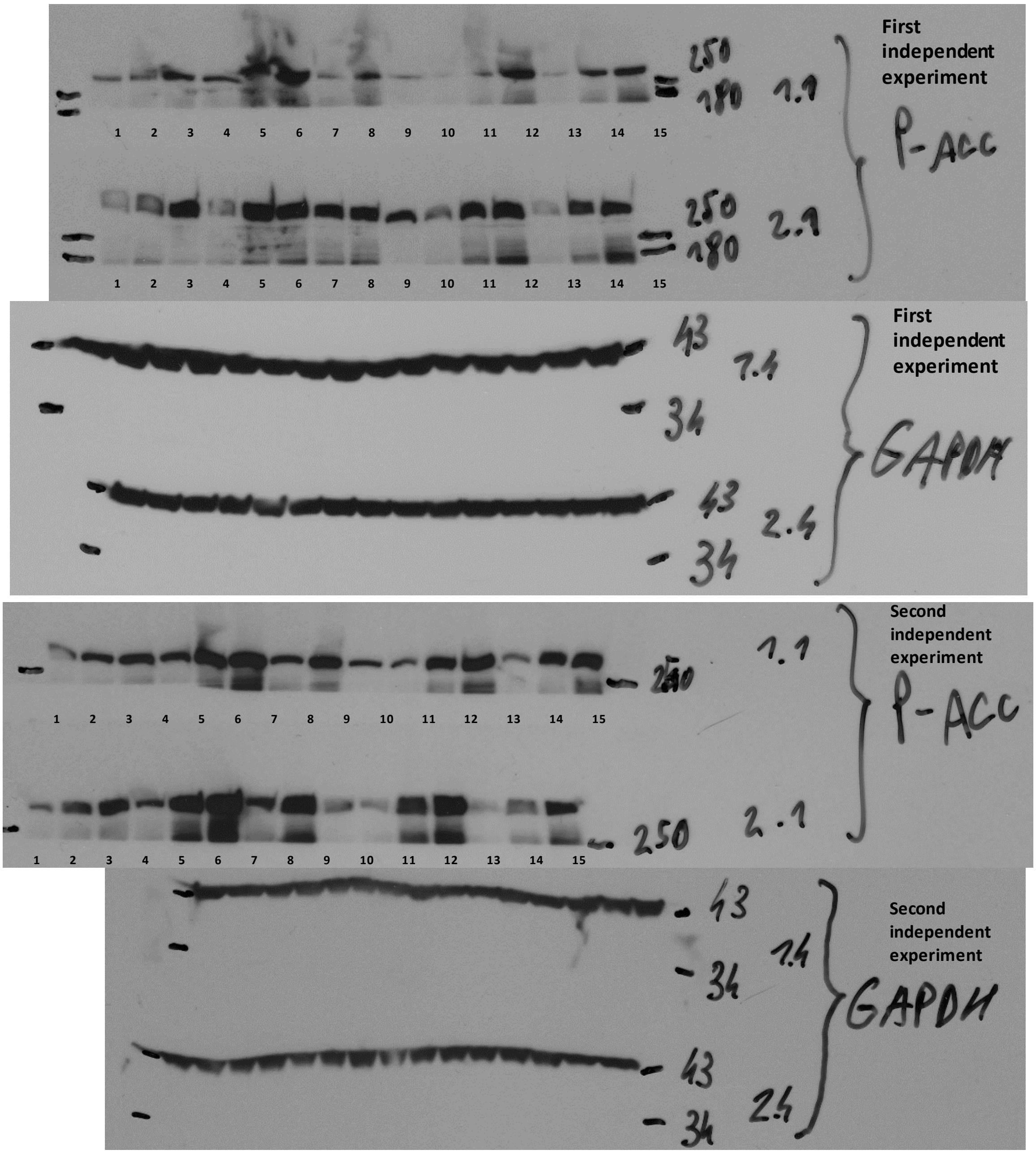

	1	2	3	4	5	6	7	8	9	10	11	12	13	14	15
**2DG (mM)**	-	0.6	4.8	-	0.6	4.8	-	-	-	-	4.8	0.6	-	4.8	0.6
**Metformin (mM)**	-	-	-	5	5	5	-	-	-	-	-	5	-	-	5
**A 769662 (µM)**	-	-	-	-	-	-	70	150	-	-	-	-	-	-	-
**Tunicamycin (ng/mL)**	-	-	-	-	-	-	-	-	50	-	-	-	-	-	-
**Mannose (mM)**	-	-	-	-	-	-	-	-	-	1	1	1	-	-	-
**Compound C (µM)**	-	-	-	-	-	-	-	-	-	-	-	-	5	5	5

Figure S11. Original western blot images (continued).

ACC phosphorylation, MDA-MB-231 cells, Figure S7I (part 2)

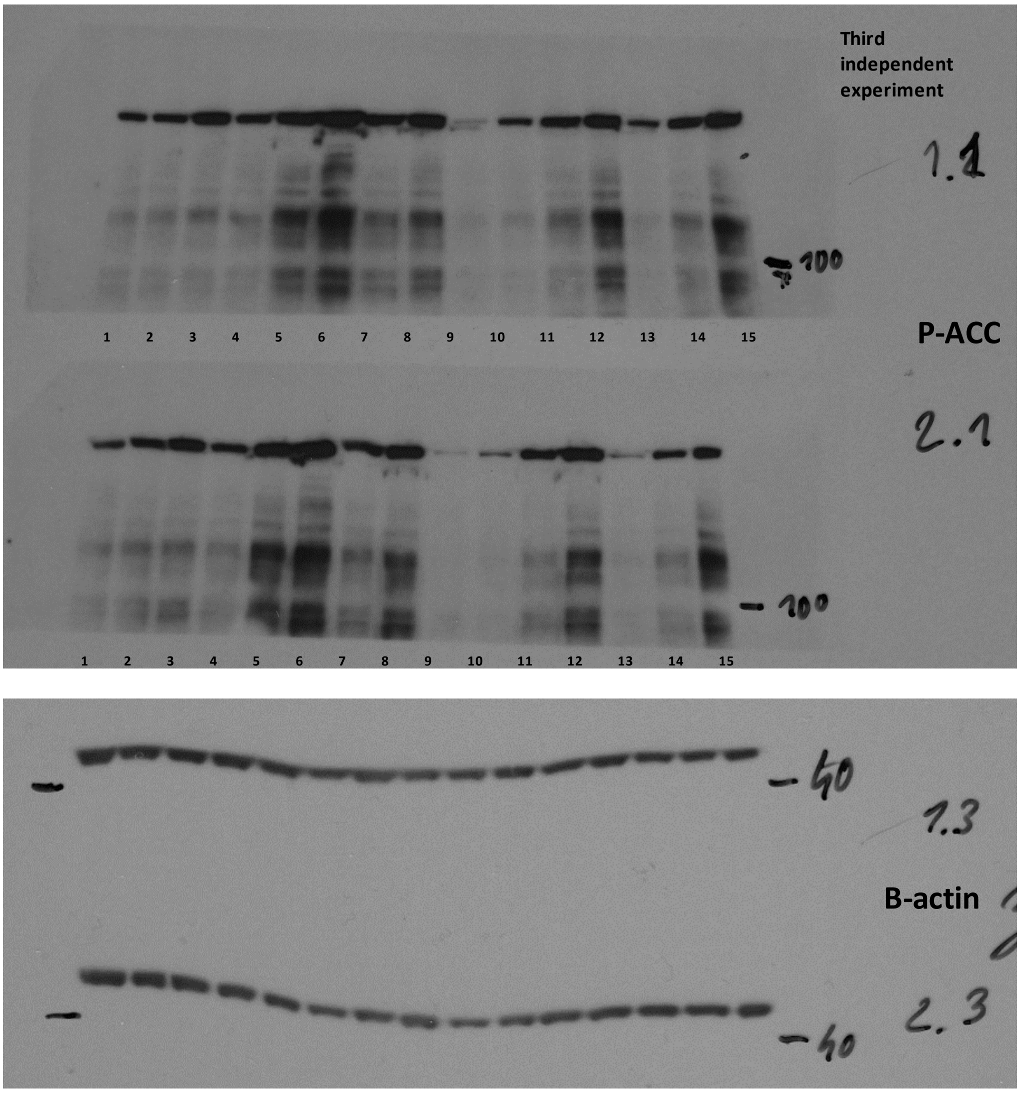

	1	2	3	4	5	6	7	8	9	10	11	12	13	14	15
**2DG (mM)**	-	0.6	4.8	-	0.6	4.8	-	-	-	-	4.8	0.6	-	4.8	0.6
**Metformin (mM)**	-	-	-	5	5	5	-	-	-	-	-	5	-	-	5
**A 769662 (µM)**	-	-	-	-	-	-	70	150	-	-	-	-	-	-	-
**Tunicamycin (ng/mL)**	-	-	-	-	-	-	-	-	50	-	-	-	-	-	-
**Mannose (mM)**	-	-	-	-	-	-	-	-	-	1	1	1	-	-	-
**Compound C (µM)**	-	-	-	-	-	-	-	-	-	-	-	-	5	5	5

Figure S11. Original western blot images (continued).

LC3B, Jurkat cells, Figure S9B & S10A (part 1)

LC3B:

Experiment 1

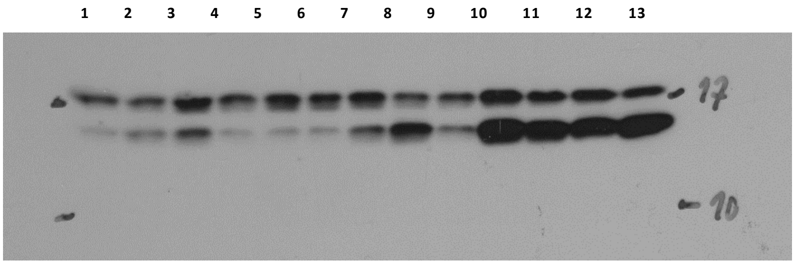

P-S6RP:

Experiment 1

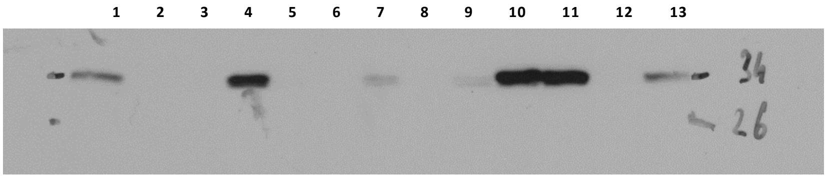

B-actin:

Experiment 1

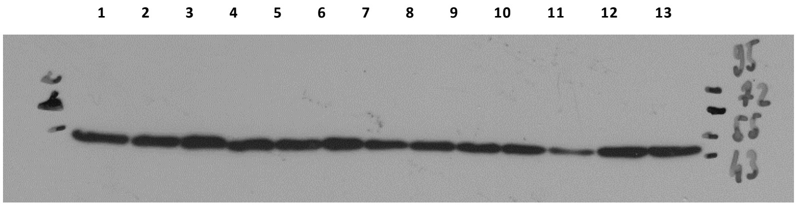

Lane	Sample
1	Control
2	0.6 mM 2DG
3	4.8 mM 2DG
4	0.3 mM Metformin
5	5 mM Metformin
6	5 mM Metformin + 0.6 mM 2DG
7	100 µM A 769662
8	1.0 µM rapamycin
9	50 ng/mL tunicamycin
10	MDA-MB-231, control
11	MDA-MB-231, control
12	MDA-MB-231, 1 µM rapamycin
13	MDA-MB-231, 50 ng/mL tunicamycin

Figure S11. Original western blot images (continued).

LC3B, Jurkat cells, Figure S9B & S10A (part 2)

LC3B:

Experiment 2

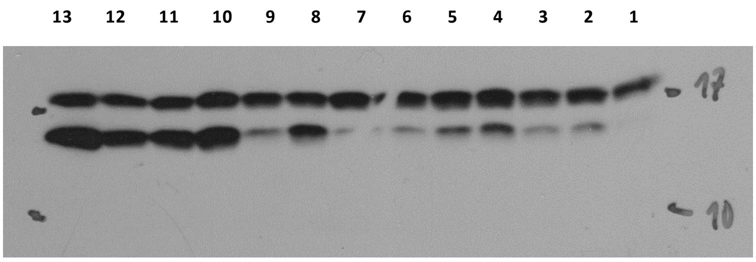

P-S6RP:

Experiment 2

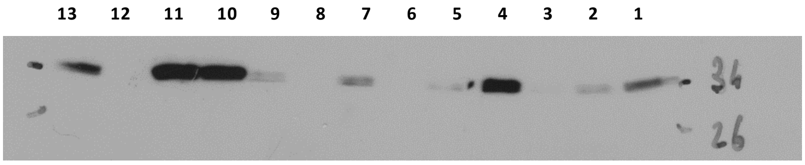

B-actin:

Experiment 2

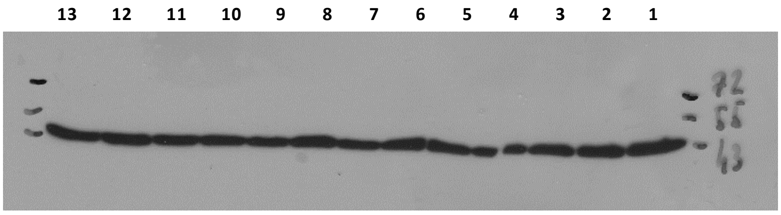

Lane	Sample
1	Control
2	0.6 mM 2DG
3	4.8 mM 2DG
4	0.3 mM Metformin
5	5 mM Metformin
6	5 mM Metformin + 0.6 mM 2DG
7	100 µM A 769662
8	1.0 µM rapamycin
9	50 ng/mL tunicamycin
10	MDA-MB-231, control
11	MDA-MB-231, control
12	MDA-MB-231, 1 µM rapamycin
13	MDA-MB-231, 50 ng/mL tunicamycin

Figure S11. Original western blot images (continued).

LC3B, Jurkat cells, Figure S9B & S10A (part 5)

LC3B:

Experiment 3

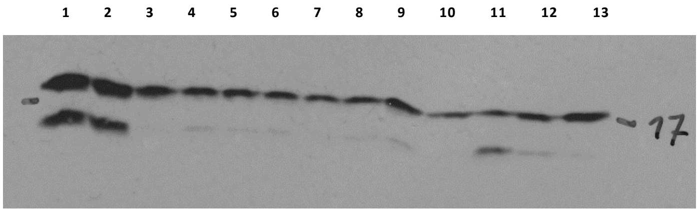

P-S6RP:

Experiment 3

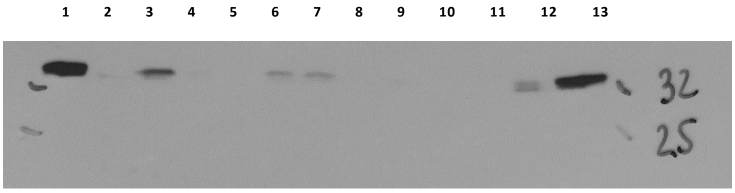

B-actin:

Experiment 3

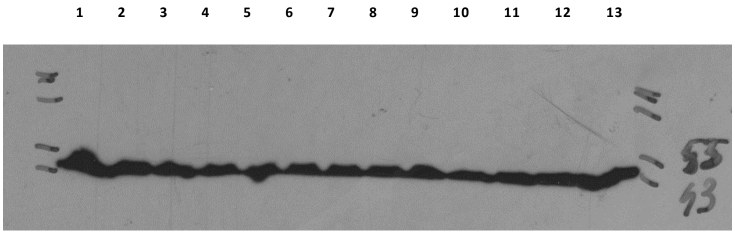

Lane	Sample
1	MDA-MB-231, Control
2	MDA-MB-231, 50 ng/mL tunicamycin
3	Control
4	0.6 mM 2DG
5	4.8 mM 2DG
6	0.3 mM Metformin
7	1 mM Metformin
8	5 mM Metformin
9	5 mM Metformin + 0.6 mM 2DG
10	100 µM A 769662
11	1.0 µM rapamycin
12	50 ng/mL tunicamycin
13	Control

Figure S11. Original western blot images (continued).
